# Bacteroides Fragilis Transplantation Reverses Reproductive Senescence by Transporting Extracellular Vesicles Through the Gut‐Ovary Axis

**DOI:** 10.1002/advs.202409740

**Published:** 2025-01-13

**Authors:** Yan Xiong, Xiaoxue Lu, Bohao Li, Shiyao Xu, Beibei Fu, Zhou Sha, Rong Tian, Rui Yao, Qian Li, Jingmin Yan, Dong Guo, Zixuan Cong, Yongliang Du, Xiaoyuan Lin, Haibo Wu

**Affiliations:** ^1^ School of Life Sciences Chongqing University Chongqing 401331 China; ^2^ Department of Clinical Microbiology and Immunology College of Pharmacy and Medical Laboratory Army Medical University (Third Military Medical University) Chongqing 400038 China; ^3^ Department of pathology Chongqing Hygeia Hospital Chongqing 401331 China

**Keywords:** bacteroides fragilis, extracellular vehicles, miR‐1246, ovarian aging, oxidative stress, SKP2

## Abstract

The diverse and dynamic population of microorganisms present in the gut microbiota may affect host health. There are evidences to support the role of gut microbiota as a key player in reproductive development. Unfortunately, the relationship between reproductive disorders caused by aging and gut microbiota remains largely unknown. Here, it is shown for the first time that gut microorganism *Bacteroides fragilis* (BF) transplantation ameliorates ovarian aging by transporting extracellular vesicles (EVs) through the gut‐ovary axis. Mechanistically, miR‐1246 is enriched in EVs derived from BF‐treated intestinal cells, and these miR‐1246‐enriched EVs are transferred to ovaries, thereby effectively improving reproductive senescence by reducing oxidative stress in the ovaries. Specifically, miR‐1246 reduces the ubiquitination of p62 and stabilizes the protein level of p62 by targeting E3 ligase SKP2. Then Keap1‐Nrf2 complex is dissociated and Keap1 is recruited to form the p62‐Keap1 complex. With the dissociation of Keap1‐Nrf2 complex, Nrf2 is released and activated, thus promoting the transcription of antioxidant enzymes and relieving reproductive senescence. Collectively, the data indicates that intestinal cell‐derived EVs serve as natural information carriers in the crosstalk between the gut and the ovary, and intestinal microorganism transplantation is a promising approach for the treatment of ovarian dysfunction diseases.

## Graphical abstract

This study demonstrates a novel mechanism by which *Bacteroides fragilis* (BF) transplantation mitigates ovarian aging via the gut‐ovary axis. Extracellular vesicles (EVs) from BF‐treated intestinal cells, enriched with miR‐1246, are transported to the ovaries, where they reduce oxidative stress by modulating the SKP2/p62/Nrf2 pathway. These findings highlight BF‐EVs as natural carriers in gut‐ovary communication, offering potential therapeutic strategies for ovarian dysfunction.

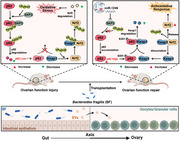

## 1 Introduction

The ovary is an essential organ for female reproduction. It produces oocytes and is the main provider of steroid sex hormones,^[^ ^1^ ^]^ therefore, the ovary is crucial for maintaining endocrine homeostasis and female fertility. The ovary is one of the human organs that exhibits early‐onset aging‐associated dysfunction, which begins to deteriorate significantly around the age of 30.^[^ ^2^, ^3^ ^]^ Female fertility decreases as a result of age‐related declines in follicle number and oocyte quality.^[^ ^4^ ^]^ The mechanisms responsible for the age‐dependent pattern of decreased ovarian function are not fully understood, but the activation of oxidative stress is considered as an important factor.^[^ ^5^, ^6^, ^7^ ^]^ Reproductive capacity is affected by redox balance, antioxidant enzymes, and oxidant enzyme levels.^[^ ^8^ ^]^ The biological development process will be disturbed due to abnormalities in oxidative stress/reactive oxygen species (ROS).^[^ ^8^ ^]^ With reproductive aging, oxidative stress/ROS levels in ovaries significantly increase.^[^ ^9^ ^]^ Modulation of oxidative stress/ROS levels may be a feasible strategy to improve ovarian aging. Currently, strategies such as using antioxidants^[^ ^10^, ^11^, ^12^, ^13^ ^]^ can alleviate ovarian oxidative stress to a certain extent. In addition, mesenchymal stem cell transplantation has become a new therapeutic strategy for restoration of reproductive function in patients with premature ovarian failure. However, due to the difficulty in acquisition, it has not been widely used in the clinic.^[^ ^14^, ^15^, ^16^ ^]^

The regulation of host health is now considered to be largely dependent on the gut microbiota.^[^ ^17^, ^18^ ^]^ The gut microbiota in healthy individuals is relatively stable and can establish a host‐bacterial mutualism.^[^ ^19^ ^]^ According to a previous report, every phase of female reproduction, including ovarian follicle and oocyte development, fertilization and embryo migration, implantation, and the whole pregnancy, even during parturition, is known to be impacted by the human microbiome.^[^ ^20^ ^]^ Since alterations in the microbiota, particularly the gut microbiota, have specific effects on the reproductive endocrine system, correction of abnormal microbiomes may improve reproductive outcomes.^[^ ^21^ ^]^ Fecal transplantation from healthy rats was reported to restore the estrous cycle and ovarian morphology while reducing androgen production in animals with polycystic ovary syndrome.^[^ ^22^ ^]^ Also, Xu et al. reported that after using fecal microbiota transplantation (FMT) to transplant feces from young mice into aging mice, the composition of the gut microbiota in FMT‐treated mice showed a “younger‐like phenotype.”^[^ ^23^ ^]^

*Bacteroides fragilis* (BF) is a commensal, gram‐negative anaerobic bacterium that is present in the human lower gastrointestinal tract^[^ ^24^, ^25^, ^26^ ^]^ and accounts for approximately 1% of the gut microbiota.^[^ ^27^, ^28^, ^29^ ^]^ It has been reported that oral administration of BF significantly increases the concentration of short‐chain fatty acids in the intestinal contents of *Salmonella*‐infected rats, which may further reduce inflammation and restore the integrity of the intestinal barrier.^[^ ^30^ ^]^ When retained in the gut, bacteria maintain a complicated and typically beneficial connection with the host. However, when the bacteria escape this environment, they can cause serious pathological conditions, such as bacteremia and abscess formation in numerous regions.^[^ ^31^ ^]^ Intestinal homeostasis is maintained by complex and dynamic interactions between the microbiome and the host.^[^ ^32^, ^33^ ^]^ BF plays an important role in maintaining intestinal homeostasis. Studies have shown that BF can restore bile acid metabolic balance through bile salt hydrolase activity and alleviate necrotizing enterocolitis by inhibiting the FXR‐NLRP3 signaling pathway.^[^ ^34^ ^]^ Furthermore, research suggests a link between the enrichment of BF in the gut and an increase lifespan.^[^ ^35^ ^]^ Thus, BF, as a probiotic, holds potential for treating diseases associated with gut microbiota dysbiosis. Moreover, reproductive system diseases‐including pregnancy complications, adverse pregnancy outcomes, PCOS, endometriosis, and cancer‐are associated with gut microbiota dysbiosis.^[^ ^20^ ^]^ We guessed that BF may play a significant role in alleviating reproductive system disorders. BF resides in the gut; however, the mechanism by which it regulates diseases related to the reproductive system to achieve inter‐organ crosstalk remains unclear. Therefore, EVs play a critical role in mediating inter‐organ communication. EVs are a variety of small vesicles with lipid bilayer membranes, and increasing evidences suggest that EVs release from host intestinal cells is a way to mediate intercellular communication.^[^ ^36^, ^37^ ^]^ EVs size varies between 20 and 500 nm, released by most cell types and can be detected in almost all body fluids.^[^ ^38^ ^]^ By transferring and exchanging biological macromolecules, including miRNAs, enclosed within the vesicles, EVs can mediate intercellular communication and regulate a variety of biological functions.^[^ ^39^, ^40^ ^]^

Considering the potential connection between gut microbiota and reproductive disorders, as well as the fact that BF is one of the most abundant probiotic in the gut microbiota. More importantly, studies have found that BF abundance is significantly lower in aging‐related atrial fibrillation compared to that in younger individuals.^[^ ^41^ ^]^ We hypothesized that BFT might relieve reproductive disorder in aging mice by improving ovarian function. In this study, we showed that BF treatment can relieve ovarian aging by transferring BF‐EVs. Delivery of BF‐EVs into ovaries alleviated ovarian oxidative stress and improved ovarian dysfunction. Mechanistically, BFT‐induced EVs transported miR‐1246 to the ovary, thereby reducing the expression of SKP2 and lowering the ubiquitination of p62, which in turn activated the Keap1‐Nrf2 antioxidant system and resulted in the alleviation of reproductive senescence. Taken together, this study provides important new insights into the involvement of gut microbiota in ovarian function and reproductive development.

## 2 Results

### 2.1 BFT Rescues Oxidative Stress Levels to Alleviate Ovarian Aging

**Figure 1:**
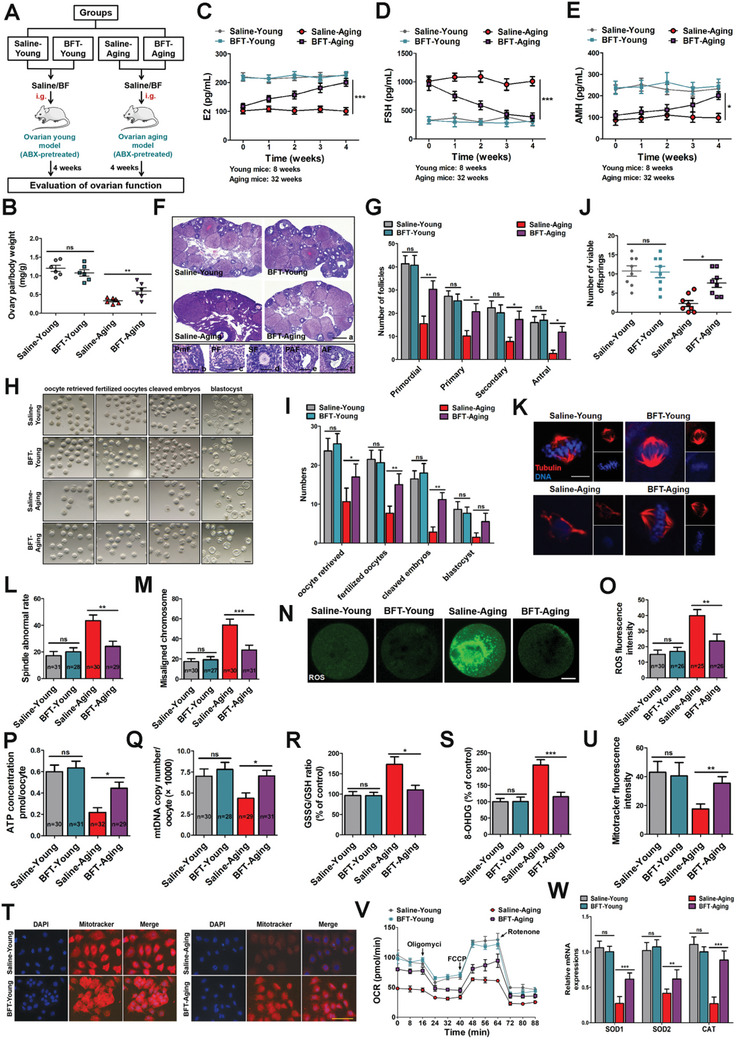
BFT rescues oxidative stress levels to relieve ovarian aging. A) Schematic diagram demonstrating the study design of in vivo experiments on BF‐relieved ovarian aging. B) Ovary‐pair ratio coefficients. (*n* = 6 in each group; one‐way ANOVA). C–E) Concentrations of E2, FSH, and AMH in Saline‐Young, BFT‐Young, Saline‐Aging, and BFT‐Aging were tested by ELISA (*n* = 6 in each group; two‐way ANOVA). *** *p* < 0.001; * *p* < 0.05. F) Histopathologic images of ovaries in the control and BFT groups. Scale bar, a, 250 mm; b, 10 mm; c, 20 mm; d, 50 mm; e, 120 mm; f, 150 mm. G) The number of follicles at different development stages (*n* = 6 in each group; two‐way ANOVA). ** *p* < 0.01; * *p* < 0.05; ns, not significant. H) Representative images of oocytes retrieved, fertilized zygotes, cleaved embryos, and blastocysts (Saline‐Young, BFT‐Young, Saline‐Aging, and BFT‐Aging). Scale bar, 100 µm. I) The number of oocytes retrieved, fertilized zygotes, cleaved embryos, and blastocysts in different groups (*n* = 6 in each group; two‐way ANOVA). ** *p* < 0.01; * *p* < 0.05; ns, not significant. J) The number of viable off‐springs conceived in different groups (*n* = 8 in each group; one‐way ANOVA). * *p* < 0.05; ns, not significant. K) Representative images of spindle assembly and chromosomes alignment in Saline and BF‐treated oocytes in young or aging mice. Oocytes were stained with anti‐tubulin antibody (red) and DAPI (blue). Representative images of normal spindle (barrel‐shaped) and chromosome alignment (toothbrush appearance) were considered normal. Scale bar, 10 µm. L) Abnormal spindle rates in different groups (*n* = 31, 28, 30, 29; one‐way ANOVA). ** *p* < 0.01; ns, not significant. M) Misaligned chromosome rates in four groups (*n* = 30, 27, 30, 31; one‐way ANOVA). *** *p* < 0.001; ns, not significant. N,O) ROS fluorescence staining (green) (N) and relative fluorescence intensity ratio (O) in MII oocytes from Saline‐Young (*n* = 30), BFT‐Young (*n* = 26), Saline‐Aging (*n* = 25) and BFT‐Aging (*n* = 26) groups (one‐way ANOVA). ** *p* < 0.01; ns, not significant. Scale bar, 20 µm. P) ATP levels were measured in different groups (*n* = 30, 31, 32, 29; one‐way ANOVA). * *p* < 0.05; ns, not significant. Q) MtDNA copy number in Saline‐Young, BFT‐Young, Saline‐Aging, and BFT‐Aging group (*n* = 30, 28, 29, 31; one‐way ANOVA). * *p* < 0.05; ns, not significant. R) GSSG/GSH ratio (*n* = 6 in each group, one‐way ANOVA). ** *p* < 0.01; ns, not significant. S) Mitochondrial oxidative stress was further confirmed by 8‐OHDG (*n* = 6 in each group, one‐way ANOVA). *** *p* < 0.001; ns, not significant. T) Mitochondrial activities were measured using MitoTracker Red. Scale bar, 60 µm. U) Quantification of mitochondrial activities (*n* = 6 in each group, one‐way ANOVA). ** *p* < 0.01; ns, not significant. V) OCR measurements were obtained. W) RT‐qPCR was performed to determine mRNA levels of oxidative stress‐related genes, including SOD1, SOD2, and CAT (*n* = 6 in each group, two‐way ANOVA). *** *p* < 0.001; * *p* < 0.05; ns, not significant.

First, we assessed the differences in ovarian function and microbial profiles between aging and young mice. Ovarian function was evaluated by examining estrogen (E2), follicle‐stimulating hormone (FSH), and anti‐mullerian hormone (AMH) concentrations, follicle counts, and embryonic development in mice from different groups. Specifically, E2 and AMH levels, follicle counts, and embryo numbers were reduced in aging mice (Figure S1A,C–E, Supporting Information), while FSH levels were elevated compared to young mice (Figure S1B, Supporting Information). More importantly, we measured BF levels in the feces of aging mice using real‐time quantitative polymerase chain reaction (RT‐qPCR) and found that BF abundance was significantly reduced in aging mice (Figure S1F, Supporting Information). We hypothesize that BF may play a positive role in regulating ovarian aging. To evaluate the potential therapeutic effects of BF administration on ovarian aging, we established a BFT mouse model by orally treating mice (ABX‐pretreated) with BF for 4 weeks (**Figure** **1**A). To confirm the effectiveness of ABX treatment and BF colonization, normal mice and germ‐free mice were used as positive and negative controls, respectively (Figure S2A, column 1 and column 4, Supporting Information). RT‐qPCR was performed to quantify bacterial 16S rDNA content in feces (Figure S2A, Supporting Information). BF levels were also examined by RT‐qPCR in different groups of ABX‐pretreated mice (Saline‐Young, BFT‐Young, Saline‐Aging, BFT‐Aging). After ABX‐pretreated mice were transplanted with BF, the relative abundance and fecal quantity of BF significantly increased in both young and aging mice (Figure S2B,C, column 2 and column 4, Supporting Information).

These data showed that BF was successfully colonized in the intestine. As shown in Figure 1B, the ovary‐ratio coefficient in the BF group was much higher than that of the control group in aging mice (column 3, column 4), and BF administration did not change in the ovary‐ratio coefficient in young mice (column 1, column 2). We also found that the concentrations of serum E2 and AMH were significantly increased in aging mice by BF treatment, while FSH level was decreased (Figure 1C–E). Further, ovaries from different groups were sectioned to evaluate follicle development. By using hematoxylin and eosin (H&E) staining, we found that aging mice exhibited a severe deterioration of follicles, and BF could effectively alleviate this deterioration (Figure 1F,G). Furthermore, the total numbers of oocytes retrieved, fertilized zygotes, cleaved embryos, and blastocysts of aging mice in the BFT group were notably elevated compared with those in the saline‐treated aging mice (Figure 1H,I). Importantly, while the number of off‐springs was not significantly affected by BF in young mice, it was increased in aging mice after BF treatment (Figure 1J). Therefore, these results suggest that BFT can partially recover ovarian function. At the same time, *Escherichia coli* (*E. coli*) and *Lactococcus lactis* (*L. lactis*) were also administrated to test the therapeutic effects on ovarian aging (Figure S3A, Supporting Information). We found that *E. coil* and *L. lactis* did not improve ovarian functions, including hormone concentrations (Figure S3B–D, Supporting Information), follicle numbers (Figure S3E,F, Supporting Information), as well as embryonic development and fertility (Figure S3G,H, Supporting Information). These results suggested that BF might be a key beneficial microbe that regulates the ovarian aging.

Previous studies have shown a correlation between the bioenergetic status of oocytes and abnormal meiotic outcomes, particularly defects in spindle positioning and chromosome scattering.^[^ ^42^ ^]^ Ovarian aging is regularly accompanied by the production of oocytes with higher rates of spindle defects and chromosomal misalignment.^[^ ^42^ ^]^ We thus tested the metaphase II spindle and chromosomal alignment of oocytes to estimate the bioenergetic status. In this model (Figure 1A), BFT group showed partially restored normal spindle appearance and inhibition of chromosomal misalignment in aging mice (Figure 1K–M). It is well known that bioenergy is closely related to cellular mitochondrial function,^[^ ^43^ ^]^ and we therefore further investigated whether BF treatment could improve the ovarian mitochondrial function in aging mice. The results showed that treatment with BF resulted in a decrease in ROS levels (Figure 1N,O), accompanied by increased ATP concentrations and mtDNA copy numbers (Figure 1P,Q), reduced GSSG/GSH ratios and 8‐OHDG levels (Figure 1R,S) and increased mitochondrial activity (Figure 1T,U). Cell viability did not significantly differ among the groups, indicating that the cells were in good condition (Figure S2D, Supporting Information). Weak Mitotracker staining ruled out poor cell condition as a factor. To further characterize the effects of BF on mitochondrial function, seahorse analysis was used to assess mitochondrial respiration in aged mice following BF transplantation. The oxygen consumption rate (OCR) of granular cells (GCs) was significantly increased in aging mice by BF treatment, relative to saline controls (Figure 1V). Similarly, oxidative stress‐related genes (SOD1, SOD2, CAT) were increased by BF treatment at the mRNA level (Figure 1W). Thus, these observations indicated that BF is able to effectively relieve ovarian aging by recovering mitochondrial function.

### 2.2 BFT Delivers EVs into the Ovary to Relieve Ovarian Aging

**Figure 2:**
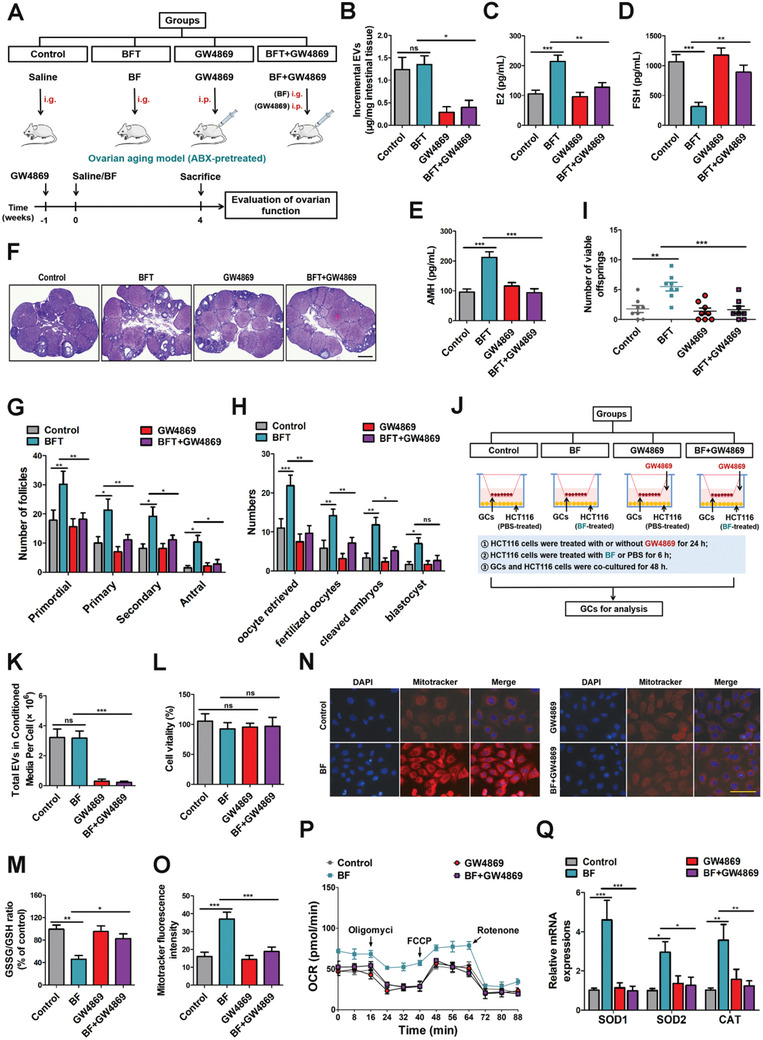
BFT delivers EVs into ovaries to relieve ovarian aging. A) Schematic diagram demonstrating the study design of in vivo experiments. B) Mice were treated with BF or BF+GW4869 respectively, and EVs were quantified (*n* = 6 in each group; one‐way ANOVA). * *p* < 0.05; ns, not significant. C–E) Concentrations of E2, FSH, and AMH in control, BFT, GW4869, and BFT + GW4869 groups (*n* = 6 in each group; one‐way ANOVA). *** *p* < 0.001; ** *p* < 0.01. F) Histopathologic images of ovaries in control, BFT, GW4869, and BFT + GW4869 groups. Scale bar, 250 mm. G) The number of follicles at different development stages (*n* = 6 in each group; two‐way ANOVA). ** *p* < 0.01; * *p* < 0.05. H) The number of oocytes retrieved, fertilized zygotes, cleaved embryos, and blastocysts in different groups (*n* = 6 in each group; two‐way ANOVA). *** *p* < 0.001; ** *p* < 0.01; * *p* < 0.05; ns, not significant. I) The number of viable offspring conceived in different groups (*n* = 8 in each group; one‐way ANOVA). *** *p* < 0.001; ** *p* < 0.01. J) A schematic overview of GCs co‐cultured with HCT116 cells, which were pre‐treated with PBS or BF. K) EVs were collected from conditioned media and quantified. L) Cell cytotoxicity was evaluated by CCK‐8 assay (*n* = 6 in each group; one‐way ANOVA). ns, not significant. M) GSSG/GSH ratio (*n* = 6 in each group, one‐way ANOVA). ** *p* < 0.01; * *p* < 0.05. N) Mitochondrial activities were measured using MitoTracker Red (scale bar, 60 µm). O) Relative fluorescence intensity of mitochondrial activities in Control, BF, GW4869, and BF + GW4869 groups (*n* = 6 in each group, one‐way ANOVA). *** *p* < 0.001. P) OCR measurements were obtained. Q) RT‐qPCR was performed to determine mRNA levels of oxidative stress‐related genes, including SOD1, SOD2, and CAT (*n* = 6 in each group, two‐way ANOVA). *** *p* < 0.001; ** *p* < 0.01.

Increasing evidences have shown that gut microbiota‐based treatment compensation is primarily attributable to the effects of intestinal cell‐derived EVs.^[^ ^36^, ^37^ ^]^ To investigate the involvement of EVs in the BF‐induced anti‐ovarian aging effects, we preincubated BF with GW4869, a neutral sphingomyelinase (nSMase) inhibitor which is able to impair EV release,^[^ ^44^, ^45^ ^]^ to interfere with the secretion of EVs by BF. As shown in Figure S4A (Supporting Information), GW4869 did not affect the vitality of BF as determined by bacterial colony counting assay. However, the production of EVs in BF was significantly inhibited after treatment with GW4869 for 4 days, as evidenced by the significant reduction in total protein content and the number of isolated EVs (Figure S4B,C, Supporting Information). We then applied GW4869 to pretreat aging mice 1 week before BF transplantation. Subsequently, aging mice were treated with saline or BF (i.g.) for 4 weeks, and ovarian function was evaluated (**Figure** **2**A). As a result, GW4869 pretreatment decreased the production of EVs from intestinal cells (Figure 2B). ELISA results showed that the application of GW4869 efficiently inhibited the effect of BFT on hormone (E2, FSH, AMH) levels (Figure 2C–E). The GW4869‐dampened therapeutic efficacy of BFT was tested in terms of ovarian function, such as number of follicles (Figure 2F,G), embryonic development, and fertility (Figure 2H,I). Next, we built an in vitro co‐culture model to examine whether EVs indeed regulate ovarian oxidative stress (Figure 2J). GW4869‐pretreated HCT116 cells (PBS/BF treated, 6 h) were co‐cultured with GCs for 48 h. It was shown that GW4869 successfully reduced EV release by HCT116 cells (Figure 2K) without affecting cell vitality (Figure 2L). Then, we analyzed the GSH levels (Figure 2M) and mitochondrial activities (Figure 2N–P) and confirmed that GW4869 pretreatment prevented BF from alleviating oxidative damage. Similar data were observed by testing oxidative stress‐related genes, and GW4869 can effectively weaken the antioxidative effects of BF treatment (Figure 2Q). Meanwhile, we observed similar results in a mouse co‐culture model (Figure S5A, Supporting Information). GW4869 effectively inhibited EV release from CT26 cells (Figure S5B, Supporting Information) while maintaining cell viability (Figure S5C, Supporting Information). EVs derived from BF‐treated CT26 cells effectively alleviated oxidative stress levels in GCs. However, GW4869 abolished the therapeutic effects of BF, including GSH levels (Figure S5D, Supporting Information), MitoTracker staining (Figure S5E,F, Supporting Information), OCR levels (Figure S5G, Supporting Information), and the expression of oxidative stress‐related genes (Figure S5H, Supporting Information). Overall, the effects of BFT were inhibited after blockade of EV generation by GW4869 preconditioning.

**Figure 3:**
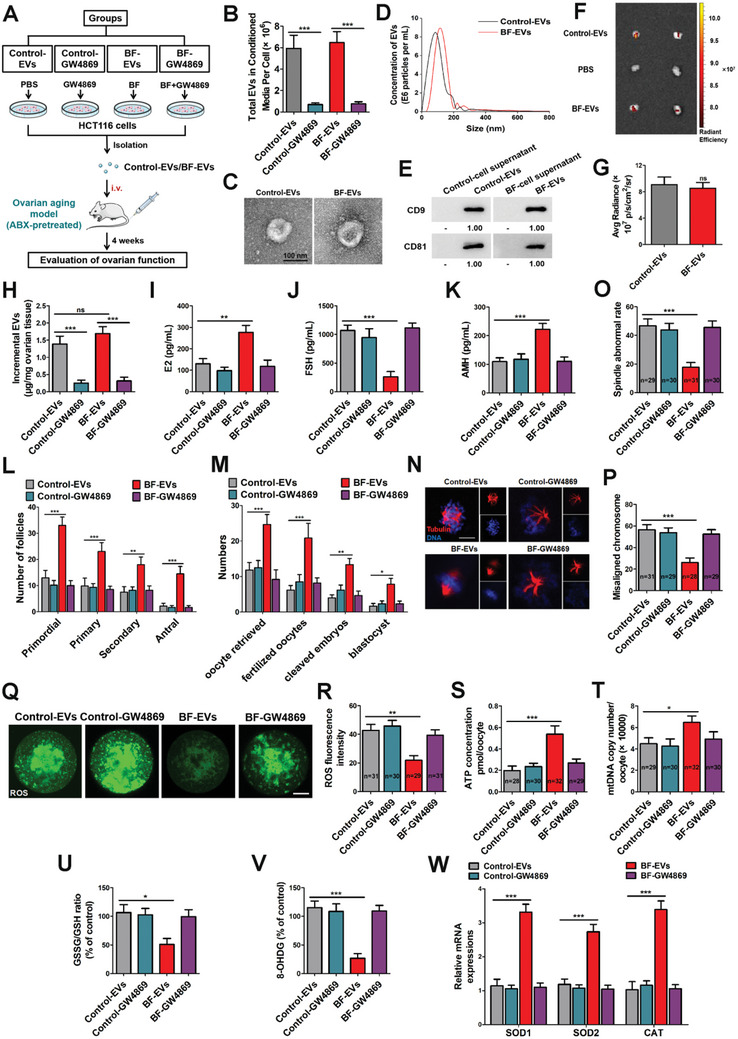
EVs derived from BF‐inoculated cell relieve ovarian aging. A) Schematic diagram demonstrating EV isolation and the study design of in vivo experiments. B) EVs were collected from cell‐conditioned media and quantified (*n* = 6 in each group, one‐way ANOVA). *** *p* < 0.001. C) Representative image of control‐EVs and BF‐EVs observed by transmission electron microscope (TEM). Scale bar, 100 nm. D) Nanoparticle tracking analysis (NTA) was performed to measure size distribution of EVs. E) Western blotting analysis of EV‐related markers CD9 and CD81. F) In vivo imaging of DiR‐labeled EVs. G) Quantified data of fluorescence signal in (F) (*n* = 6, *t*‐test). ns, not significant. H) Mice were treated with EVs from cultured HCT116 cells, followed by EV quantification (*n* = 6 in each group, *t*‐test). ns, not significant. I–K) The levels of E2, FSH, and AMH were measured by ELISA in different groups (*n* = 6 in each group, one‐way ANOVA). *** *p* < 0.001; ** *p* < 0.01. L) The number of follicles at different development stages in Control‐EVs, Control‐GW4869, BF‐EVs, and BF‐GW4869 groups (*n* = 6 in each group, two‐way ANOVA). *** *p* < 0.001; ** *p* < 0.01. M) The number of oocytes retrieved, fertilized zygotes, cleaved embryos, and blastocysts in Control‐EVs, Control‐GW4869, BF‐EVs, and BF‐GW4869 groups (*n* = 6 in each group, two‐way ANOVA). *** *p* < 0.001; ** *p* < 0.01; ** *p* < 0.01. N) Representative images of spindle assembly and chromosomes alignment in different treated oocytes. Oocytes were stained with anti‐tubulin antibody (red) and DAPI (blue). Scale bar, 10 µm. O) Abnormal spindle rates in Control‐EVs, Control‐GW4869, BF‐EVs, and BF‐GW4869 groups (*n* = 29, 30, 31, 30; one‐way ANOVA). *** *p* < 0.001. P) Misaligned chromosome rates in Control‐EVs, Control‐GW4869, BF‐EVs and BF‐GW4869 groups (*n* = 31, 29, 28, 29; one‐way ANOVA). *** *p* < 0.001. Q,R) ROS fluorescence staining (green) (Q) and relative fluorescence intensity ratio (R) in MII oocytes from Control‐EVs (*n* = 31), Control‐GW4869 (*n* = 30), BF‐EVs (*n* = 29) and BF‐GW4869 (*n* = 31) groups (one‐way ANOVA). ** *p* < 0.01. Scale bar, 20 µm. S) ATP levels were measured in Control‐EVs, Control‐GW4869, BF‐EVs, and BF‐GW4869 treated oocytes (*n* = 28, 30, 32, 29; one‐way ANOVA). *** *p* < 0.001. T) MtDNA copy number in Control‐EVs, Control‐GW4869, BF‐EVs, and BF‐GW4869 groups (*n* = 29, 30, 32, 30; one‐way ANOVA). * *p* < 0.05. U) GSSG/GSH ratio (*n* = 6 in each group, one‐way ANOVA). * *p* < 0.05. V) Mitochondrial oxidative stress was further confirmed by 8‐OHDG (*n* = 6 in each group, one‐way ANOVA). *** *p* < 0.001. (W) RT‐qPCR was performed to determine mRNA levels of oxidative stress‐related genes, including SOD1, SOD2, and CAT (*n* = 6 in each group, two‐way ANOVA). *** *p* < 0.001.

To directly evaluate the effects of EVs, we collected EVs from cultured HCT116 cells and established an EV treatment model (**Figure** **3**A). In brief, HCT116 cells were pretreated with PBS or GW4869 for 24 h, followed by treatment with PBS or BF for 6 h. The EVs were then isolated from the cell culture medium for the four groups: Control‐EVs, Control‐GW4869, BF‐EVs, and BF‐GW4869. We confirmed that EV release in this assay was markedly reduced by GW4869 (Figure 3B, column 2 and column 4), and verified their identity as EVs according to morphological characteristics and specific markers (Figure 3C–E). In this model, we showed that EVs could be directly delivered into ovaries (Figure 3F,G). Although the quantity of EVs delivered into the ovaries was not considerably different between BF‐EVs and control‐EVs (Figure 3H), data on hormone (E2, FSH, AMH) levels (Figure 3I–K), follicle number (Figure 3L), and embryonic development (Figure 3M) indicated that aging mouse ovarian function was significantly restored in the BF‐EVs group. Moreover, the proportion of oocytes contained chromosomal misalignment was lower in BF‐EVs treatment group than that in control group, suggesting that BF‐EVs promote normal meiotic outcomes (Figure 3N–P). We next investigated whether BF‐EVs treatment would produce antioxidative phenotypes similar to BFT. We found that treatment with BF‐EVs markedly decreased ROS levels (Figure 3Q,R), and increased ATP and mtDNA levels (Figure 3S,T) in aging oocytes. Also, supplementation with BF‐EVs in aging mice altered ovarian mitochondrial function, including the GSH ratio (Figure 3U), 8‐OHDG (Figure 3V), and oxidative stress‐related gene levels (Figure 3W). Taken together, these findings indicated that BF‐EVs effectively relieve ovarian aging by recovering mitochondrial function.

Next, we used mouse primary intestinal epithelial cells (IECs) as another cell model to confirm our findings (Figure S4D, Supporting Information). The results showed that GW4869 effectively inhibited EV generation in primary IECs (Figure S4E, Supporting Information). As expected, BF‐IECs‐EVs significantly reduced oxidative stress levels in ovarian aging mice (Figure S4F–H, Supporting Information). Meanwhile, the embryonic development and fertility defects of aging mice were markedly rescued by BF‐IECs‐EVs treatment (Figure S4I,J, Supporting Information), indicating that BF‐IECs‐EVs could relieve ovarian aging. Collectively, these results indicated that BFT can deliver intestinal cell‐derived EVs into the ovary, thereby alleviating ovarian aging.

### 2.3 BFT Delivers miR‐1246 into the Ovary Through Intestinal Cell‐Derived EVs

EVs contain abundant miRNAs that participate in the intercellular communications.^[^ ^46^, ^47^ ^]^ We hypothesize that miRNAs enclosed within EVs play a crucial role in alleviating ovarian aging.

**Figure 4:**
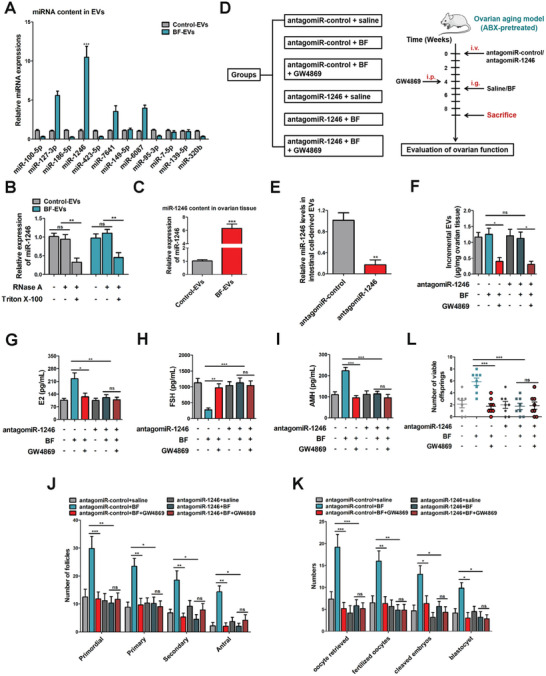
BFT can deliver miR‐1246 through EVs into ovaries. A) RT‐qPCR was performed to determine miRNA levels in EVs from different groups (*n* = 6, two‐way ANOVA). *** *p* < 0.001. B) HCT116 cells were treated with RNase A with or without Triton X‐100 after PBS/BF treatment, followed by RT‐qPCR analysis (*n* = 6, one‐way ANOVA). ** *p* < 0.01; ns, not significant. C) RT‐qPCR was performed to determine miRNA levels in ovarian tissue (*n* = 6, two‐way ANOVA). *** *p* < 0.001. D) Schematic diagram demonstrating the study design of in vivo experiments. E) The level of miR‐1246 was measured by RT‐qPCR (*n* = 6 in each group; one‐way ANOVA). ** *p* < 0.01. F) Mice were treated with antagomiR‐1246 or antagomiR‐control, and EVs were quantified (*n* = 6 in each group; one‐way ANOVA). * *p* < 0.05; ns, not significant. G–I) The levels of E2, FSH, and AMH were measured by ELISA in different groups (*n* = 6 in each group, one‐way ANOVA). *** *p* < 0.001; ** *p* < 0.01; * *p* < 0.05; ns, not significant. J) The number of follicles at different development stages in different groups (*n* = 6 in each group, two‐way ANOVA). *** *p* < 0.001; ** *p* < 0.01; * *p* < 0.05; ns, not significant. K) The number of oocytes retrieved, fertilized zygotes, cleaved embryos, and blastocysts in the antagomiR‐control + saline, antagomiR‐control + BF, antagomiR‐control + BF + GW4869, antagomiR‐1246 + saline, antagomiR‐1246 + BF, and antagomiR‐1246 + BF + GW4869 groups (*n* = 6 in each group, two‐way ANOVA). *** *p* < 0.001; ** *p* < 0.01; * *p* < 0.05; ns, not significant. L)The number of viable off‐springs conceived (*n* = 8 in each group; one‐way ANOVA). *** *p* < 0.001; ns, not significant.

In this study, we selected twelve candidate miRNAs (miR‐100‐5p, miR‐127‐3p, miR‐186‐5p, miR‐1246, miR‐423‐5p, miR‐7641, miR‐149‐5p, miR‐6087, miR‐95‐3p, miR‐7‐5p, miR‐139‐5p, and miR‐320b) from the miRNA profiles of BF‐EVs and control‐EVs for further investigation.^[^ ^48^ ^]^ Following prior research, we maintained consistent treatment methods for HCT116 cells. Briefly, HCT116 cells were treated with either BF or PBS for 6 h. EVs were then isolated from the cell culture supernatant, which were referred to as BF‐EVs and control‐EVs. The collected EVs were subjected to high‐throughput miRNA sequencing, generating robust data to support our hypothesis. The data showed that the RNA level of miR‐1246 was significantly enriched in BF‐EVs (**Figure** **4**A). Then, we asked whether miR‐1246 was packaged into the EVs. RT‐qPCR analysis showed that miR‐1246 level was significantly down‐regulated in both control‐EVs and BF‐EVs in response to RNase A and Triton X‐100 simultaneous treatment, but was rarely altered upon RNase A treatment alone (Figure 4B), indicating that extracellular miR‐1246 was mainly encapsulated in EVs instead of being released directly. In the EV treatment model, miR‐1246 was also highly expressed in ovarian tissue after treatment with BF‐EVs (Figure 4C). These results indicated that the EVs mediate the transfer of miR‐1246 into ovaries. To further evaluate the effectiveness of BF treatment in other tissues, miR‐1246 levels were measured in the liver. We found that miR‐1246 levels increased following BF‐EV treatment (Figure S3I, Supporting Information). Therefore, we propose that the BF‐regulated gut‐liver axis may also play a key role in liver diseases.

Then, miR‐1246 knockout was performed to examine the effect of miR‐1246 on ovarian function (Figure S6A, Supporting Information). The miR‐1246 knockout efficiency was validated in Figure S6B (Supporting Information). Ovarian function was tested by assessing hormone levels, number of follicles, and embryonic development. We found that miR‐1246 knockout EVs failed to recover the hormone (E2, FSH, AMH) levels in aging mice (Figure S6C–E, Supporting Information). Similar results were obtained by testing number of follicles (Figure S6F,G, Supporting Information), embryonic development, and number of off‐springs (Figure S6H,I, Supporting Information). Moreover, we built a miR‐1246 knockdown mouse model by tail vein injection of antagomiR‐1246 to further confirm our findings (Figure 4D). The injection successfully caused the reduction of miR‐1246 level in intestinal cell‐derived EVs (Figure 4E). Mice were orally treated with BF after pretreatment with GW4869 for 1 week. The production of EVs was effectively inhibited by GW4869 pretreatment (Figure 4F). We found that when miR‐1246 level in EVs decreased, the therapeutic effects of BFT on ovarian aging were significantly dampened (Figure 4G–L), which is consistent with the data collected from EVs blockade (GW4869 treatment), suggesting that the functional role of BFT probably depends on the miR‐1246 encapsulated in EVs.

### 2.4 miR‐1246 Enriched‐EVs Relieve Oxidative Stress

**Figure 5:**
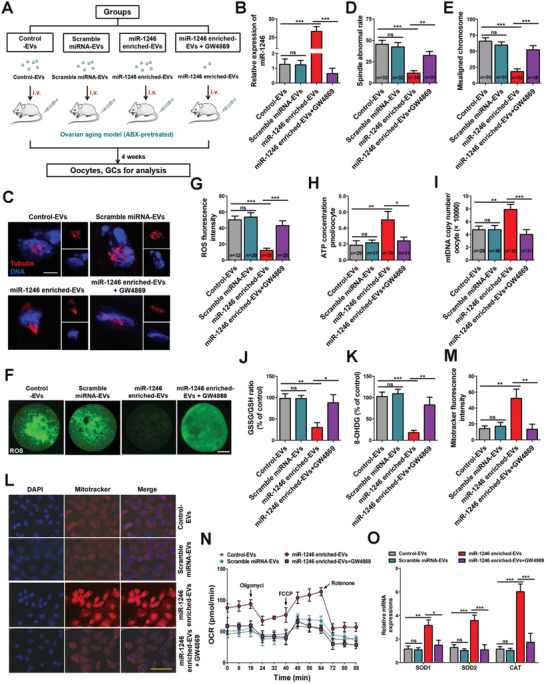
miR‐1246 enriched‐EVs relieve oxidative stress. A) Schematic diagram demonstrating the study design of in vivo experiments. B) miR‐1246 levels in EVs were tested by RT‐qPCR (*n* = 6 in each group, one‐way ANOVA). *** *p* < 0.001; ns, not significant. C) Representative images of spindle assembly and chromosomes alignment in Control‐EVs, Scramble miRNA‐EVs, miR‐1246 enriched‐EVs, and miR‐1246 enriched‐EVs+GW4869 treated oocytes. Oocytes were stained with anti‐tubulin antibody (red) and DAPI (blue). Scale bar, 10 µm. D) Abnormal spindle rates in Control‐EVs, Scramble miRNA‐EVs, miR‐1246 enriched‐EVs, and miR‐1246 enriched‐EVs+GW4869 groups (*n* = 32, 32, 30, 31; one‐way ANOVA). *** *p* < 0.001; ** *p* < 0.01; ns, not significant. E) Misaligned chromosome rates in Control‐EVs, Scramble miRNA‐EVs, miR‐1246 enriched‐EVs, and miR‐1246 enriched‐EVs+GW4869 groups (*n* = 30, 31, 32, 28; one‐way ANOVA). *** *p* < 0.001; ns, not significant. F,G) ROS fluorescence staining (green) (F) and relative fluorescence intensity ratio (G) in MII oocytes from Control (*n* = 32), Scramble miRNA (*n* = 29), miR‐1246 enriched‐EVs (*n* = 30) and miR‐1246 enriched‐EVs+GW4869 (*n* = 28) groups (one‐way ANOVA). *** *p* < 0.001; ns, not significant. Scale bar, 20 µm. H) ATP levels were measured in Control‐EVs, Scramble miRNA‐EVs, miR‐1246 enriched‐EVs, and miR‐1246 enriched‐EVs+GW4869 treated oocytes (*n* = 29, 31, 30, 31; one‐way ANOVA). ** *p* < 0.01; * *p* < 0.05; ns, not significant. I) MtDNA copy number in Control‐EVs, Scramble miRNA‐EVs, miR‐1246 enriched‐EVs, and miR‐1246 enriched‐EVs+GW4869 groups (*n* = 29, 30, 32, 31; one‐way ANOVA). *** *p* < 0.001; ** *p* < 0.01; ns, not significant. J) GSSG/GSH ratio (*n* = 6 in each group, one‐way ANOVA). ** *p* < 0.01; * *p* < 0.05; ns, not significant. K) Mitochondrial oxidative stress was further confirmed by 8‐OHDG (*n* = 6 in each group, one‐way ANOVA). *** *p* < 0.001; ** *p* < 0.01; ns, not significant. L) Mitochondrial activities were measured using MitoTracker Red. Scale bar, 60 µm. M) Quantification of mitochondrial activities (*n* = 6 in each group, one‐way ANOVA). ** *p* < 0.01; ns, not significant. N) OCR measurements were obtained. O) RT‐qPCR was performed to determine mRNA levels of oxidative stress‐related genes, including SOD1, SOD2, and CAT (*n* = 6 in each group, two‐way ANOVA). *** *p* < 0.001; ** *p* < 0.01; * *p* < 0.05; ns, not significant.

To further study the biological effects of miR‐1246 in BF‐EVs, we isolated miR‐1246 enriched‐EVs from the spent culture medium of HCT116 cells transfected with miR‐1246 mimics. Additionally, to explore the interaction between EVs and miR‐1246, we established a GW4869 treatment group. Specifically, HCT116 cells were pretreated with GW4869 for 24 h before being transfected with miR‐1246 mimics, and EVs were then collected from the culture medium and referred to as the miR‐1246 enriched‐EVs + GW4869 group. Mice were administered with different EVs (control‐EVs, Scramble miRNA‐EVs, miR‐1246 enriched‐EVs, miR‐1246 enriched‐EVs+GW4869) via tail vein injection once a week for 4 weeks (**Figure** **5**A). The effectiveness of miR‐1246 enrichment was confirmed by RT‐qPCR (Figure 5B). We showed that miR‐1246 enriched‐EVs significantly decreased abnormal meiotic outcomes and chromosome misalignment in ovarian aging mice (Figure 5C–E). Further, overexpression of miR‐1246 in EVs significantly reduced the ROS level (Figure 5F,G), GSH level (Figure 5J), and 8‐OHDG level (Figure 5K), while increasing the ATP and mtDNA levels (Figure 5H,I) and strengthening mitochondrial function (Figure 5L,M). The OCR was significantly increased by miR‐1246 enriched‐EVs (Figure 5N). In addition, the mRNA levels of antioxidant enzymes were markedly increased by supplementation with miR‐1246 enriched‐EVs (Figure 5O). More importantly, GW4869 eliminated the biological effects of miR‐1246 enriched‐EVs. Together, these results indicate that miR‐1246 effectively alleviates ovarian aging by decreasing oxidative stress, suggesting that the function of miR‐1246 depends on EV delivery.

### 2.5 miR‐1246 Targets SKP2 to Regulate the p62/Keap1/Nrf2 Pathway

**Figure 6:**
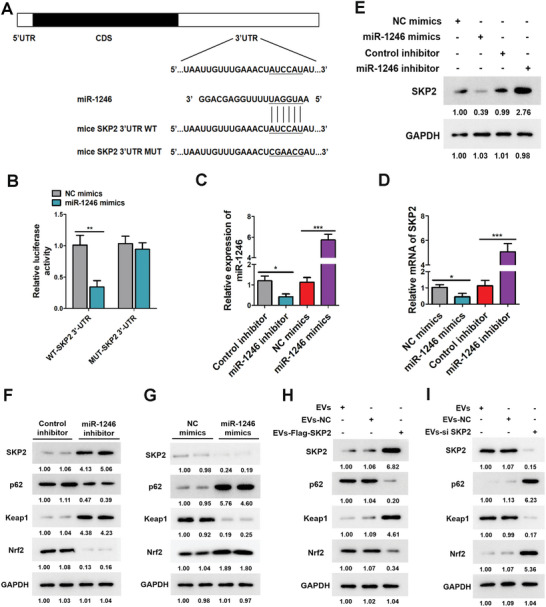
miR‐1246 targets SKP2 to regulate the p62/Keap1/Nrf2 pathway. A) The binding site of miR‐1246 and SKP2 in the Starbase database. B) The binding relationship between miR‐1246 and SKP2 was confirmed using dual‐luciferase assay (*n* = 3, one‐way ANOVA). ** *p* < 0.01. C) After transfection of Control inhibitor, miR‐1246 inhibitor, NC mimics, and miR‐1246 mimics into GCs, miR‐1246 expression was determined using RT‐qPCR (*n* = 3, one‐way ANOVA). *** *p* < 0.001, * *p* < 0.05. D) SKP2 mRNA expression was determined using RT‐qPCR (*n* = 3, one‐way ANOVA). *** *p* < 0.001, * *p* < 0.05. E) GCs were transfected with miR‐1246 mimic or inhibitor or the corresponding scrambled control. Western blotting was used to detect the protein level of SKP2. F) GCs were transfected with miR‐1246 inhibitor or the corresponding scrambled control. Western blotting was used to detect the protein levels of p62, Keap1, and Nrf2. G) GCs were transfected with miR‐1246 mimic or the corresponding scrambled control. Western blotting was used to detect the protein levels of p62, Keap1, and Nrf2. H) GCs were co‐cultured with EVs (overexpression of SKP2), the SKP2, p62, Keap1, and Nrf2 protein expressions were analyzed by western blotting. I) GCs were co‐cultured with EVs (knockdown of SKP2), the SKP2, p62, Keap1, and Nrf2 protein expressions were analyzed by western blotting. The blots represented three independent experiments.

To explore the underlying mechanism of miR‐1246 regulating ovarian function, we used an online tool (targetscan.org) to predict the downstream targets of miR‐1246, and SKP2 was selected as a candidate target (**Figure** **6**A). Luciferase vectors containing the wild‐type or mutant 3′‐untranslated region (UTR) sequence of SKP2 were constructed. We found that miR‐1246 mimic significantly decreased the relative luciferase activity of the wild‐type vectors, while the luciferase activity of mutant vectors was not altered (Figure 6B). Next, miR‐1246 mimics and inhibitor were used to investigate the role of miR‐1246. The effectiveness of mimics and inhibitor was verified by RT‐qPCR (Figure 6C). The results showed that miR‐1246 mimics significantly reduced the mRNA and protein levels of SKP2 (Figure 6D,E). According to reports, the Nrf2‐Keap1 system is currently considered as one of the main cellular defense mechanisms against oxidative stress.^[^ ^49^ ^]^ The accumulation of p62 forms a p62‐Keap1 complex, which leads to the persistent activation of Nrf2.^[^ ^50^, ^51^ ^]^ In our study, transfection of miR‐1246 inhibitor into GCs resulted in a decrease in p62 protein level (Figure 6F). Conversely, transfection of miR‐1246 mimics resulted in increased expression of p62 and further activated the Keap1/Nrf2 pathway (Figure 6G). Intestinal cells were transfected with Flag‐SKP2 or SKP2 siRNA, respectively, then EVs were isolated and added into the culture medium of GCs. We found that with the increase of SKP2 expression in EVs, p62 protein level decreased in GCs, accompanied by the inhibition of Keap1‐Nrf2 signaling (Figure 6H). These results were also validated by SKP2 knockdown in EVs (Figure 6I).

We next asked how miR‐1246 enriched‐EVs mediate the accumulation of p62 in GCs. The cellular level of proteins is determined by the balance between their synthesis and degradation rates, including mRNA stability, transcription, and translation.^[^ ^52^, ^53^ ^]^ We showed that the addition of miR‐1246 enriched‐EVs did not affect the promoter activities or mRNA levels of p62 in WT and SKP2 knockdown GCs (Figure S7A,B, Supporting Information), indicating that the change of p62 protein levels might be due to degradation process. Proteasomal and lysosomal pathways are the two main mechanisms for protein degradation in eukaryotic cells.^[^ ^54^ ^]^ Subsequently, GCs were pretreated with MG132 (proteasomal degradation inhibitor) or E64d + PepA (lysosomal degradation inhibitor), respectively. Compared with control group, p62 protein was significantly increased in the MG132‐treated cells (Figure S7C, lane 1 and lane 2, Supporting Information). The increase in p62 protein level caused by MG132 treatment was also observed in the miR‐1246 enriched‐EVs group (Figure S7C, lane 3 and lane 4, Supporting Information). However, this difference was not shown between the E64d + PepA treatment and the control group (Figure S7D, Supporting Information). We further examined the ubiquitination levels of p62 among different groups. We found that the protein level of p62 was increased in EV‐treated group compared with control group (Figure S7E, lane 1 and lane 3, Supporting Information), while when cells were pretreated with MG132, both the protein and ubiquitination levels of p62 accumulated (Figure S7E, lane 1 and lane 2, Supporting Information). These results further revealed that p62 underwent ubiquitination, and this modification was regulated by EVs. Importantly, the effect of miR‐1246‐enriched EVs on p62 degradation disappeared in si‐SKP2 cells (Figure S7C–E, Supporting Information), indicating that SKP2 is involved in the proteasomal degradation of p62.

### 2.6 SKP2 Interacts with p62 and Promotes p62 Ubiquitination at K48

**Figure 7:**
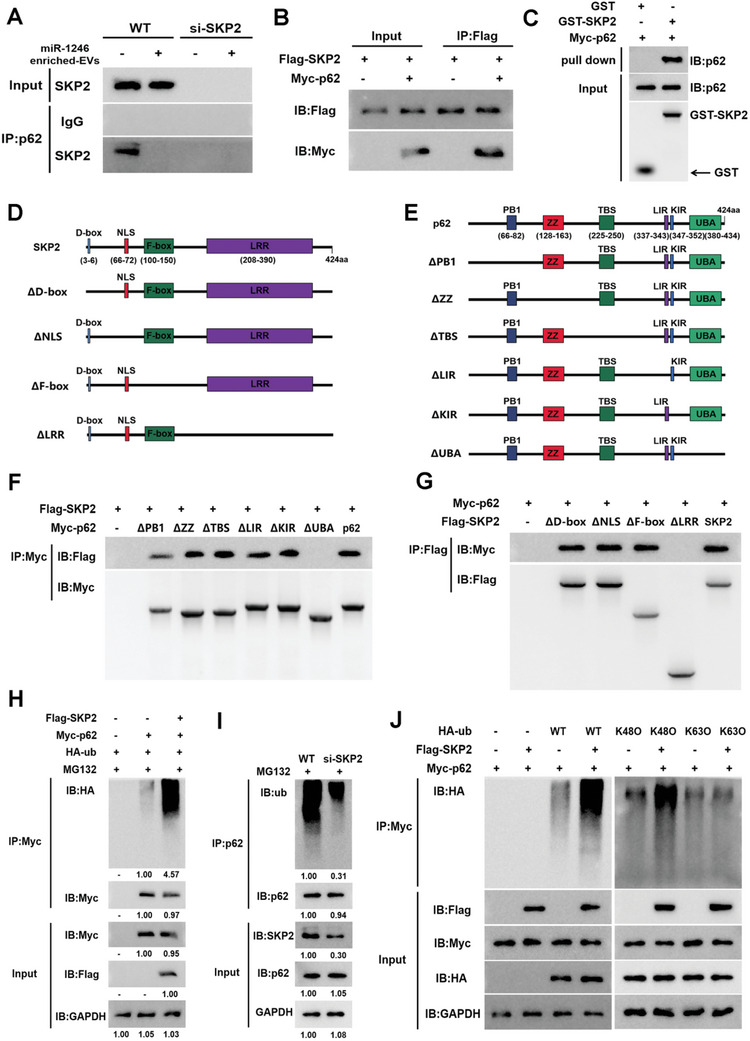
SKP2 interacts with p62 and promotes p62 ubiquitination at K48. A) GCs (WT, si‐SKP2) were treated with or without miR‐1246 enriched‐EVs for 24 h. The interaction between SKP2 and p62 was detected by co‐IP. B) Flag‐labeled SKP2 and Myc‐labeled p62 were transfected into GCs (WT). And the interaction between SKP2 and p62 was detected by co‐IP. C) The interaction between SKP2 and p62 was detected by GST pulldown assay in vitro. D,E) Schematic diagram of the SKP2 and p62 truncations. F) Interaction between the p62 truncations and SKP2 in GCs was detected using co‐IP. G) Interaction between the SKP2 truncations and p62 in GCs was detected using co‐IP. H) Flag‐SKP2 and Myc‐p62 plasmids were transfected into GCs, and the ubiquitination of p62 was detected. I) Ubiquitination of p62 was analyzed in WT and si‐SKP2 GCs. J) GCs were transfected with ubiquitin mutant (K48O, K63O), Myc‐labeled p62, or Flag‐labeled SKP2 as indicated. The ubiquitination of p62 was detected. Blots were representative of three independent experiments.

To determine whether SKP2 directly interacts with p62, we performed coimmunoprecipitation (co‐IP) of insoluble fractions with anti‐p62 and anti‐SKP2 antibodies. The interaction between endogenous SKP2 and p62 was confirmed in WT GCs (**Figure** **7**A). The interaction between exogenous SKP2 and p62 was also validated by transfection of Flag‐SKP2 and Myc‐p62 plasmids in GCs (Figure 7B). Further, the direct interaction between SKP2 and p62 was confirmed through GST‐pulled down assay (Figure 7C). Next, we constructed truncation mutants to identify the interaction domain between p62 and SKP2 (Figure 7D,E). Flag‐labeled SKP2 was effectively pulled down by truncated p62 mutants, except for the mutant lacking the UBA domain (Figure 7F), and p62 can be pulled down by SKP2 truncations, except for the mutant lacking the LRR domain (Figure 7G), suggesting that the UBA domain of p62 and the LRR domain of SKP2 are essential for the interaction. Further, Flag‐labeled SKP2 and Myc‐labeled p62 were transfected into GCs, and co‐IP results showed that SKP2 enhanced the ubiquitination of p62 (Figure 7H). On the contrary, when SKP2 was inhibited by siRNA, the ubiquitination of p62 was decreased (Figure 7I). Moreover, by using ubiquitin mutants that are only capable of forming either K48 or K63 linkages, we found that SKP2 specifically induced K48‐linked ubiquitination of p62 (Figure 7J). Taken together, our data showed that SKP2 interacted with p62 and promoted the ubiquitination of p62 at K48, thereby leading to the degradation of p62.

Our previous data showed that the accumulation of p62 activates the Keap1‐Nrf2 pathway (Figure 6I). To understand the specific mechanism of miR‐1246 regulating the Keap1/Nrf2‐dependent antioxidative response, miR‐1246 enriched‐EVs were isolated and co‐cultured with GCs for 48 h. The results showed that Keap1 protein level was decreased and Nrf2 protein level was elevated in GCs with miR‐1246 enriched‐EVs treatment (Figure S8A, Supporting Information). By using co‐IP and western blot analysis, we showed that in the control group, Keap1 was able to bind to Nrf2 (Figure S8B, Supporting Information), thereby leading to the ubiquitination of Nrf2 (Figure S8C, Supporting Information); however, in the miR‐1246 enriched‐EVs group, the Keap1‐Nrf2 complex was dissociated and Keap1 was recruited to form the p62‐Keap1 complex (Figure S8B, Supporting Information), which, according to previous reports, was the main reason for the decrease in Keap1 protein levels.^[^ ^55^ ^]^ Since p62 regulated the Keap1‐Nrf2 interaction through p62 phosphorylation at Ser351, thus controlling the Nrf2 antioxidative stress response.^[^ ^56^ ^]^ We showed that the phosphorylation of p62 was indeed upregulated by miR‐1246 enriched‐EVs treatment (Figure S8D, Supporting Information). Furthermore, we confirmed this mechanism in GCs by using the p62 S351A mutant, which failed to interact with Keap1 (Figure S8E, Supporting Information). These results collectively suggested that p62 accumulation caused by miR‐1246 enriched‐EVs treatment is important for the stability of the p62‐Keap1 complex, which disrupts the Keap1‐Nrf2 association and promotes Nrf2 activation. More importantly, clinical samples would strengthen the conclusions of this study by providing robust validation of the findings. It was found that NRF2 is expressed at low levels in ovarian cancer patients but is highly expressed in healthy individuals, as shown in the TCGA database (Figure S4K, Supporting Information). Although we do not have direct clinical sample data, the NRF2 data in ovarian cancer provide indirect evidence supporting the important role of the NRF2 pathway in the antioxidant capacity of ovarian tissue. These database findings support the biological rationale of this study, indicating that NRF2 activation has a protective effect on ovarian health. We suggest that future clinical research could validate whether Bacteroides fragilis can regulate human ovarian health through the NRF2 pathway.

### 2.7 p62/Keap1/Nrf2 Signaling Plays a Vital Role in Regulating Ovarian Aging

**Figure 8:**
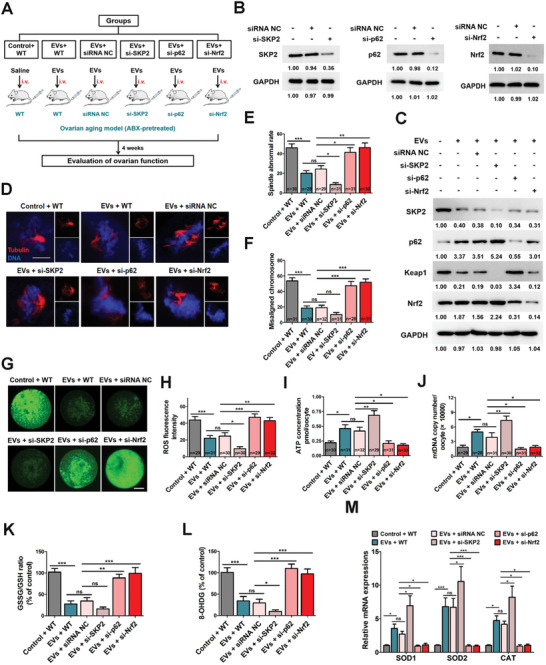
SKP2 plays a vital role in regulating p62/Keap1/Nrf2 pathway. A) Schematic diagram demonstrating the study design of in vivo knockdown experiments on BF‐EVs‐relieved ovarian aging. B) The SKP2, p62, and Nrf2 protein expressions were analyzed by western blotting. C) GCs were collected from different groups of mice for protein extraction, and western blotting was performed to detect the protein levels of SKP2, p62, Keap1, and Nrf2. D) Representative images of spindle assembly and chromosomes alignment in different groups. Oocytes were stained with anti‐tubulin antibody (red) and DAPI (blue). Representative images of normal spindle (barrel‐shaped) and chromosome alignment (toothbrush appearance) were considered normal. Scale bar, 10 µm. E) Abnormal spindle rates in different groups (*n* = 30, 28, 29, 31, 31, 30; one‐way ANOVA). *** *p* < 0.001; ** *p* < 0.01; ns, not significant. F) Misaligned chromosome rates in six groups (*n* = 31, 30, 32, 31, 28, 31; one‐way ANOVA). *** *p* < 0.001; ns, not significant. G,H) ROS fluorescence staining (green) (G) and relative fluorescence intensity ratio (H) (*n* = 29, 31, 30, 30, 29, 32. one‐way ANOVA). *** *p* < 0.001; ** *p* < 0.01; * *p* < 0.05; ns, not significant. Scale bar, 20 µm. I) ATP levels were measured in different groups (*n* = 30, 31, 32, 29, 31, 30; one‐way ANOVA). ** *p* < 0.01; * *p* < 0.05; ns, not significant. J) MtDNA copy number in Control‐WT, EVs‐WT, EVs‐siRNA NC, EVs‐si‐SKP2, EVs‐si‐p62, and EVs‐si‐Nrf2 groups (*n* = 29, 28, 31, 30, 31, 32; one‐way ANOVA). ** *p* < 0.01; * *p* < 0.05; ns, not significant. K) GSSG/GSH ratio. (*n* = 6 in each group, one‐way ANOVA). *** *p* < 0.001; ** *p* < 0.01; ns, not significant. L) Mitochondrial oxidative stress was further confirmed by 8‐OHDG (*n* = 6 in each group, one‐way ANOVA). *** *p* < 0.001; * *p* < 0.05; ns, not significant. M) RT‐qPCR was performed to determine mRNA levels of oxidative stress‐related genes, including SOD1, SOD2, and CAT (*n* = 6 in each group, two‐way ANOVA). *** *p* < 0.001; * *p* < 0.05; ns, not significant. Blots were representative of three independent experiments.

To evaluate the potential importance of p62/Keap1/Nrf2 pathway in ovarian aging in vivo, we established knockdown mouse models by using an in vivo transfection method. BF‐IECs‐EVs were transferred into knockdown mice (WT, siRNA NC, si‐SKP2, si‐p62, si‐Nrf2) via tail vein injection once a week for 4 weeks, and then samples were collected to evaluate ovarian function (**Figure** **8**A). The efficiencies of knockdowns and relative expressions of SKP2/p62/Keap1/Nrf2 in each group were confirmed by western blotting (Figure 8B,C). We showed that EVs ameliorated ovarian oxidative stress levels as well as meiotic arrest (Figure 8D–M), which is consistent with our previous findings (Figures 1, 2, 3). Importantly, we found that EVs failed to improve abnormal meiotic outcomes (Figure 8D–F) or relieve oxidative stress levels in p62 or Nrf2 knockdown mice (Figure 8G–M). Meanwhile, knockdown of SKP2 showed significant additive effects on EVs treatment in most experiments (Figure 8D–M). These data collectively indicated that the reversal of reproductive senescence by BF‐IECs‐EVs relies on the p62/Keap1/Nrf2 pathway.

## 3 Discussion

The gut microbiota and humans coexist in a harmonious balance.^[^ ^57^ ^]^ Once this balance is disrupted by external factors, humans will experience a variety of adverse physiological reactions.^[^ ^57^, ^58^ ^]^ The balance between the gut microbiota and humans can be re‐established by microbiota transplantation, which can effectively restore various physiological functions.^[^ ^59^ ^]^ Gut microbiota remodeling was reported to systemically rescue dysregulated bile acid homeostasis.^[^ ^60^ ^]^ Moreover, fecal microbiota transplantation remarkably restored the gut microbial community, thus ameliorating gastrointestinal dysfunction and motor deficits in mice with PD.^[^ ^61^ ^]^ Reproductive diseases, including abnormal pregnancy outcomes, PCOS, endometriosis, and cancer, may be caused by imbalances in the composition of the gut microbiota.^[^ ^20^ ^]^ More importantly, Bacteroides was the most abundant bacteria,^[^ ^24^ ^]^ suggesting that it may play an important role in reproductive diseases. BF is the representative of Bacteroides.^[^ ^24^ ^]^ It has been reported that BF may influence reproductive‐related diseases, such as intrahepatic cholestasis of pregnancy.^[^ ^62^ ^]^ Thus, BF demonstrates therapeutic potential in the treatment of reproductive system diseases. Ovarian aging is the focus of our current investigation into reproductive system diseases. Notably, our results revealed that BF levels were lower in aging mice compared to young mice. As a probiotic, we hypothesized that BF may play a positive role in the treatment of ovarian aging. In this study, we confirmed this hypothesis. Through in vitro and in vivo models, we showed that BF‐treated intestinal cells can transfer EVs to the ovary, thereby alleviating ovarian aging, and identified that BF‐EVs act as a messenger in communication between the gut and the ovary.

Intestinal mucosal cells are constantly exposed to millions of microbes, which have a major impact on physiological functions of proximal and distal cell by controlling intestinal homeostasis.^[^ ^36^ ^]^ EVs released by gut bacteria or host intestinal cells mediate intra‐ and interkingdom crosstalk.^[^ ^36^ ^]^ Recently, intestinal cell‐derived EVs have been demonstrated to promote intestinal inflammation and cancer by the BF/miR‐149‐3p pathway.^[^ ^48^ ^]^ Therefore, targeting the BF/miR‐149‐3p pathway is a promising strategy for treating individuals with intestinal inflammation and colorectal cancer. ROS has an important role in the reproductive processes, including oocyte quality, ovulation, fertilization, embryo implantation, and embryo development. In this study, we found that BF‐treated intestinal cell‐derived EVs ameliorated oxidative stress, and further improved the quality of oocytes, implying that BFT may increase fertilization rate, pregnancy rate, and fertility.^[^ ^63^, ^64^, ^65^, ^66^ ^]^ Our study reveals that BF‐EVs recover meiotic outcomes of aging oocytes. Mitochondria are the major generators of ROS, an imbalance in ROS can lead to impairment of their function.^[^ ^5^ ^]^ We found that after 4 weeks of treatment with BF‐EVs, ROS levels were significantly reduced, and ATP and mtDNA levels were markedly increased. Mitochondrion are the core of cellular energy metabolism and the site of most ATP generation.^[^ ^67^ ^]^ Aberrations in ATP production can also lead to mitochondrial dysfunction. In our study, we strongly demonstrated that BF‐EV treatment effectively restores mitochondrial function in the ovaries, while also reducing ovarian oxidative stress and further improving ovarian aging. Importantly, BF‐EVs treatment effectively improved the fertility of mice. Our findings provide a potential therapeutic basis for overcoming reproductive disorders caused by ovarian aging.

**Figure 9:**
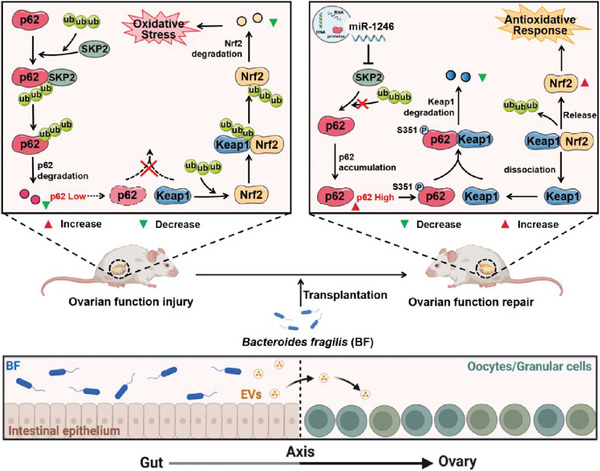
Flowchart of BFT ameliorating ovarian aging by transporting EVs containing miR‐1246, which inhibits the expression of SKP2 and promotes p62 accumulation. This process improves reproductive senescence by reducing oxidative stress in the ovaries.

Overall, we have established a new relationship between the gut and reproductive system, namely the gut‐ovary axis, through which EVs from BF‐treated intestinal cells can be transferred to ovaries and alleviate ovarian aging. Further, we identified that miR‐1246 plays an essential role in the impact of BF‐EVs on reproductive function. miRNAs play a crucial role in intercellular communication. As regulators of gene expression, they influence protein expression by binding to target mRNAs, thereby modulating various cellular physiological functions.^[^ ^68^ ^]^ Mechanistically, miR‐1246 encapsulated in the EVs inhibits the proteasomal degradation of p62 by targeting E3 ligase SKP2. The accumulation of p62 stabilizes the p62‐Keap1 complex, thereby disrupting the Keap1‐Nrf2 association and exerting an antioxidant effect by releasing Nrf2 (**Figure** **9**). More importantly, miR‐1246 within EVs plays a critical role in regulating oxidative stress in the ovaries, with EVs serving as carriers in this process. This finding highlights the strong association between miR‐1246 and ovarian aging. Our study addresses a research gap regarding the relationship between intestinal cell‐derived EVs and ovarian aging, with a particular focus on the role of miRNAs in this process. In conclusion, these findings not only provide new insights into the treatment of age‐related reproductive diseases by improving gut microbiota, but also determine the important role of BF‐EVs in the gut‐ovary axis.

## 4 Experimental Section

### Ethics Statement

This study was carried out in strict accordance with the Guidelines for the Care and Use of Animals of Chongqing University. Animal experimental procedures were approved by the Laboratory Animal Welfare and Ethics Committee of Chongqing University (CQU‐IACUC‐RE‐202209‐002).

### Cell and Bacteria Culture

HCT116 cells (CCL‐247) and CT26 cells (CRL‐2638) were obtained from the American Type Culture Collection (ATCC, Manassas, VA). Cells were cultured in RPMI‐1640 medium (Gibco, San Jose, CA, USA) containing 10% (v/v) fetal bovine serum (Gibco). All cells were kept in a humidified incubator with CO_2_ at 37 °C. The BF strain ATCC 43 860, *L. lactis* strain NZ9000, *E. coli* strain MG1655 were used in this study. BF was grown in brain‐heart infusion (BHI) broth anaerobically at 37 °C with a gas mix of 5% H_2_, 10% CO_2_, and 85% N_2_. BHI was supplemented with 0.0005% hemin and 0.5 µg mL^−1^ vitamin K1 for optimal growth. *L. lactis* was cultured at 30 °C under static conditions in M17 medium supplemented with 1% glucose. *E. coli* was cultured in LB medium at 37 °C.

### BF Treatment into Cells

First, BF (10^7^ CFUs per 100 mL medium) was cultured in complete medium containing GW4869 (10 × 10^−6^ m) for 4 days, and the viability of BF was assessed by bacterial colony counting assay on BHI agar plates. In this study, BF was pretreated with GW4869 to avoid the influence of BF‐derived EVs. Then, HCT116 cells were exposed to BF in penicillin/streptomycin‐free RPMI‐1640 for 6 h. After 6 h, the bacterial strain‐containing media was swapped out for conventional cell culture medium.

### EV Isolation

EVs were isolated using an Extraction Kit (Umibio, Shanghai, China) in accordance with the manufacturer's instructions. In brief, after reaching 80–90% confluence of HCT116 cells (WT, miR‐1246 KO), serum‐free medium was added for 48 h to avoid vesicle contamination from serum. The conditioned medium was collected and centrifuged at 3000 g for 10 min to remove cells and debris. EV concentrate solution was added proportionally to the supernatant and stood at 4 °C for 2 h, followed by centrifugation at 10 000 g for 2 h at 4 °C. Subsequently, the precipitate, enriched with EVs, was obtained and resuspended in PBS. All isolated EVs were further quantified according to protein content using the BCA Protein Assay Kit (Thermo Fisher Scientific, 23227) following the manufacturer's instructions.^[^ ^69^ ^]^

Intestinal EVs were isolated by differential centrifugation based on a previously described method.^[^ ^70^ ^]^ The isolated intestinal tissues were grounded in PBS, and then enzymatically digested for 2 h with DMEM medium containing type II collagenase (1 mg mL^−1^; Gibco, 17101015). Pieces of intestinal tissue containing supernatant were centrifuged at 300 g for 10 min at 4 °C. The supernatant was collected and centrifuged at 3000 g for 25 min and 10 000 g for 60 min. The supernatant was collected, filtered twice through a 0.22 µm sterile filter (Beyotime, FF342), and then ultra‐centrifuged at 100 000 g for 1 h. EVs were washed with 30 mL sterile PBS and then centrifuged at 100 000 g for 1 h. The final pellets were resuspended in PBS.

### Primary IEC Isolation and Culture

The isolation of IECs was conducted as described by Inmaculada López‐Almela et al.^[^ ^71^ ^]^ In brief, the small intestine was washed with cold PBS, opened longitudinally, and cut into small pieces. To isolate the epithelium, the tissue was incubated twice in Hansk´s balanced salt solution with calcium and magnesium (HBSS, Yuanpei, Shanghai, B430KJ) containing 5 mm EDTA (Thermo Fisher Scientific, 17892), 1 mm DTT, 100 µg mL^−1^ streptomycin and 100 U mL^−1^ penicillin (Beyotime, C0222) for 30 min at 37 °C with orbital shaking. Following each incubation, supernatant fractions were filtered using 100 µm nylon cell strainers (Biosharp, BS‐100‐CS) and centrifuged to harvest cell suspensions.

### Antibiotic Treatment

In order to deplete the gut microbiota, mice were treated with broad‐spectrum antibiotics (ABX) (1 g L^−1^ streptomycin; 1 g L^−1^ ampicillin; 1 g L^−1^ mg L^−1^ gentamicin, 0.5 g L^−1^ vancomycin) in drinking water for 1 week as described.^[^ ^72^ ^]^ The ABX was refreshed every 2 days to maintain its potency, and the cages were changed daily to ensure sterility.

### Animal Experiments

All young (8 weeks), aging (32 weeks) female ICR mice and germ‐free mice used in this study were purchased from Hunan SJA Laboratory Animal Co., Ltd. Additionally, male mice (12–13 weeks old) were obtained from the same source. For BFT administration, mice were administered with saline or BF (i.g., 10^9^ CFU/0.2 mL, every day) for 4 weeks. In terms of other microbial transplantation administration, mice were administered with *E. coli* or *L. lactis* (i.g., 10^9^ CFU/0.2 mL, every day) for 4 weeks.

For administration of an EV secretion inhibitor, GW4869 was given via intraperitoneal injection (i.p.; every day, 1 week) at a concentration of 1.25 mg kg^−1^. GW4869 pre‐treated mice was then administered with saline or BF (GW4869‐pretreated BF, i.g., 10^9^ CFU/0.2 mL, every day, 4 weeks). GW4869 was continuously administered to the mice until they were killed for further study.

In the antagomiR inhibition of miR‐1246 assay, mice were randomly divided into two groups: antagomiR‐control and antagomiR‐1246. Mice were injected with either antagomiR‐control or antagomiR‐1246 (i.v.; 10 mg kg^−1^ in 200 µL saline, once a week for 4 weeks). The effectiveness of knockdown was confirmed by RT‐qPCR. Subsequently, knockdown mice were treated with saline or BF (GW4869‐pretreated BF, i.g., 10^9^ CFU/0.2 mL, every day, 4 weeks). After treatment, the mice were euthanized, plasma and ovarian tissue were collected.

### Knockdown Mouse Model

In vivo gene knockdown was performed as previously described.^[^ ^73^ ^]^ Briefly, siRNA (40 µg) was combined with the in vivo‐jetPEI delivery reagent (Polyplus‐transfection, NY, USA) in a 5% glucose solution (N/P ratio = 8). The solution was mixed and incubated at room temperature for 30 min and were then intraperitoneally injected into mice.

The siRNA sequences are listed as follows: si‐SKP2: AUCACAAAGUCUUUGUCACUC; si‐p62: UGUAGAUGCGGAAGAUGUCAU; si‐Nrf2: GCAGGACAUGGAUUUGAUUTT.

Knockdown efficiency in ovary tissue was detected by western blotting.

### In Vivo Imaging

DiR‐labeled EVs (200 µL) were injected via the tail vein. Four weeks after injection, mice were euthanized, and ovarian tissue was collected. Fluorescence images were captured using a fluorescence imaging system to ascertain EV distribution.

### Treatment with miR‐1246 Enriched‐EVs

miR‐1246 mimics were transfected into HCT116 cells using Lipofectamine 3000 (Invitrogen). Fresh RPMI‐1640 medium (1% penicillin/streptomycin, 1% l‐glutamine, 10% EV‐depleted FBS) was subsequently used instead of transfection medium (Thermo Fisher Scientific, CA, USA). After 48 h of culture, the used medium was collected for EV isolation. The Extraction Kit was used to isolate EVs from HCT116 cells medium following the manufacturer's instructions. RT‐qPCR was used to measure the quantity of miR‐1246 in EVs.

### Transmission Electron Microscopy (TEM)

Fresh EVs (20 µL) were added to a carbon‐coated copper electron microscope grid and negatively stained with a phosphotungstic acid solution (Sigma–Aldrich) for 5 min. The transmission electron microscope was used to capture and analyze images (HITACHI, Tokyo, Japan).

### Nanoparticle Tracking Analysis

The extracellular vesicle solution was repeatedly blown to evenly distribute EVs. Subsequently, the size distribution of vesicles was directly determined by NTA using a NanoSight LM10 (Malvern Instrument Ltd., Malvern, UK).

### Isolation of Primary GCs and Oocytes

Aging female mice were superovulated via intraperitoneal injection of 10 IU pregnant mare serum gonadotropin (PMSG, Zhejiang, China) after BF/BF‐EVs (WT/miR‐1246 KO) treatment, followed by injection of 10 IU human chorionic gonadotropin (hCG, Sansheng, Ningbo, Zhejiang, China) 48 h later. The cumulus‐oocyte complexes were collected from the ampulla portion of the oviduct under a stereomicroscope. GCs were removed by a brief incubation in 1 mg mL^−1^ hyaluronidase. Oocytes and GCs were obtained, respectively. The oocytes were then cultured in M16 medium (Sigma–Aldrich), and GCs were cultured in DMEM:F12 (1:1) medium containing 15% fetal bovine serum at 37 °C with 5% CO_2_ for further analysis.

### OCR Analysis

OCR was measured using a seahorse XF24 analyzer (Seahorse bioscience). Briefly, GCs (4 × 10^4^ cells per well) were seeded in XFe24 Cell Culture Microplates. For OCR measurement, cells were covered with 500 µL assay medium (XF base medium (Seahorse, 102353), 1 mm sodium pyruvate, 1 mm L‐glutamine, and 10 mm glucose). Port injections were performed with 1 mm oligomycin, 3 mm FCCP, 0.5 mm antimycin, and rotenone.

### Co‐Culture of HCT116 Cells with Mouse GCs

For co‐culture experiments, HCT116 cells were inoculated with BF or PBS for 6 h. Then HCT116 cells were co‐cultured with GCs for 48 h. In inhibition experiments, HCT116 cells were pretreated with EVs‐free media containing 10 µm GW4869 (Umibio) for 24 h. Subsequently, BF was administered to the GW4869‐pretreated‐HCT116 cells for 6 h. Finally, GCs and HCT116 cells were co‐cultured for 48 h. GCs were used for study.

### Ovarian Follicle Counting

After being treated with BF/EVs for 4 weeks, mice were euthanized. Subsequently, ovaries were extracted, fixed in 4% formalin, and observed with HE staining. The counts of primordial, primary, secondary, and antral follicles were determined by selecting three typical sections from each ovary. All experiments were conducted six times. The results were presented as the mean ± standard deviation, and *p* < 0.05 was considered statistically significant.

### Embryo Culture

Fifteen female mice in each group (control and BFT/EVs group) were housed with male mice for 21 days after treatment (2–3 female mice and 1 male mouse were housed in the same cage). The female mouse was separated once it was confirmed that a copulatory plug was present. The counts of oocytes retrieved, fertilized oocytes, cleaved embryos, blastocysts in each group were recorded.

### Measurement of E2, FSH, and AMH Levels

After BF/EV treatment for 4 weeks, mouse plasma was obtained to evaluate the expression levels of E2, FSH, and AMH using an ELISA Kit (Mybiosource, USA) following the manufacturer's guidelines. In brief, serum samples were added to each well and incubated at 37 °C for 30 min. Then, the wells were washed three times with wash buffer and HRP‐conjugate reagent was added and incubated at 37 °C for 60 min. The wells were then washed three times with wash buffer and substrate A and B solutions were added. The mixture was incubated at 37 °C for 15 min. Finally, a stop solution was used to halt the reaction and measured the light absorbance using a spectrophotometer.

### Immunofluorescence

Oocytes were permeabilized in Enhanced Immunostaining Permeabilization Buffer (Beyotime, P0097) at room temperature for 15 min after being fixed in 4% paraformaldehyde for at least 30 min. Subsequently, the oocytes were incubated overnight at 4 °C with an anti‐tubulin antibody (1:1000, Abcam, Cambridge, UK, ab6160), following a blocking step with QuickBlockTM Blocking Buffer for immunostaining (Beyotime, P0260) for 8 h. The oocytes were then washed and treated with corresponding secondary antibodies at room temperature for 2 h. After 10 min of DAPI counterstaining, the oocytes were examined under a confocal fluorescence microscope.

### Measurement of ROS Levels

An ROS Assay Kit was utilized to measure ROS levels (Beyotime, Shanghai, China). Briefly, oocytes were exposed to dichlorofluorescein (DCFH), an oxidation‐sensitive fluorescent probe, at 37 °C for 30 min before observation under a fluorescence microscope.

### Evaluation of Total ATP Content

ATP levels in oocytes were determined using the bioluminescent somatic cell assay kit (Sigma, MO, USA). In brief, 20 oocytes were pooled and processed in accordance with the manufacturer's instructions. Each test comprised a six‐point standard curve (0, 0.1, 0.5, 1.0, 10, and 50 pmol of ATP), from which the ATP content was calculated using the formula obtained through linear regression analysis of the curve.

### Estimation of mtDNA Copy Numbers

The lysis buffer, comprising 50 mm Tris, 0.1 mm EDTA, 100 g mL^−1^ Proteinase K, and 0.5% Tween‐20, was added to a single GV or MII oocyte and placed in a PCR tube. The oocyte was incubated at 55 °C for 30 min, followed by 95 °C for 10 min. PCR products were amplified with mtDNA‐specific primers and ligated into a T‐vector to obtain purified DNA.

### Measurement of GSH and 8‐OHDG Levels

GCs were exposed to 10 µm 4‐chloromethyl‐6.8‐difluoro‐7‐hydroxycoumarin (CMF2HC, Invitrogen) for 30 min prior to the measurement of GSH levels using a fluorescence microscope. The 8‐OHDG levels were assessed using ELISA (Chundu, Wuhan, China, CD20013) in accordance with the manufacturer's instructions. In brief, 10 µL samples of diluted plasma (1:4) were mixed with 8‐OHDG monoclonal antibody solution and HRP‐conjugate reagent, and incubated at 37 °C for 60 min, followed by washing for three times with wash buffer. Subsequently, substrate A and B solution were added and incubated at 37 °C for 15 min. Finally, the reaction was stopped using stop solution, and the light absorbance was measured with a spectrophotometer.

### MitoTracker Assays

Following the manufacturer's instructions, MitoTracker Red CMXRos (Beyotime, C1049B) was used to measure the mitochondrial activity in GCs treated with BF or EVs. Briefly, the MitoTracker product was diluted to a final concentration of 1 mm using anhydrous dimethylsulfoxide (DMSO). From the stock solution, a final working concentration of 200 nm was prepared by dilution in DMEM/F12. GCs were cultured in a 12‐well plate and treated with 200 nm MitoTracker red dye at 37 °C for 30 min. Subsequently, the cells underwent two washes in PBS and were fixed overnight at 4 °C using a fixative. After a 10‐min counterstaining with DAPI, the GCs were examined using a fluorescence microscope.

### Reverse Transcription and RT‐qPCR

The fecal DNA was extracted according to the instructions of the Stool Genomic DNA Extraction Kit (Solarbio). The isolated DNA was then subjected to RT‐qPCR analysis to quantify changes in bacterial load in the samples. And BF was also detected through RT‐qPCR assay. The reaction protocol was as follows: 95 °C for 2 min; 40 cycles at 95 °C for 15 s, and 60 °C for 30 s.

RNA extraction was performed following previous methods.^[^ ^74^ ^]^ Quantification of miR‐1246 was performed using a SuperScript III One‐Step RT‐PCR kit (Thermo Fisher Scientific). The relative expression of miR‐1246 was normalized by U6 and calculated using the 2^−ΔΔCT^ method. In brief, this procedure included a 30 s pre‐incubation at 95 °C followed by 40 cycles of denaturation at 95 °C for 5 s and annealing at 60 °C for 30 s.

The primers used in the study are listed as follows (5′‐3′): 16S rDNA V3‐V4 (F): TCCTACGGGAGGCAGCAGT, 16S rDNA V3‐V4 (R): GGACTACCAGGGTATCTAATCCTGTT; BF 16s (F): TGGACTGCAACTGACACTGA, BF 16s (R): GCCGCTTACTGTATATCGCA. miR‐1246 (F): TTCGACGTGAATGGATTTTTG, miR‐1246 (R): TATCGTTGTACTCCAGACCAAGAC; U6 (F): CTCGCTTCGGCAGCACA, U6 (R): AACGCTTCACGAATTTGCGT.

### Luciferase Reporter Assays

StarBase (https://starbase.sysu.edu.cn/) was utilized to predict the binding site of miR‐1246 and SKP2. The SKP2 sequence containing the wild‐type (WT) and mutant‐type (MUT) binding site was cloned into the pGL3 reporter gene vector (Promega). The SKP2 WT or SKP2 MUT reporter vectors were co‐transfected with either the miR‐1246 mimic or NC mimic into GCs using Lipofectamine 3000 (Invitrogen). The dual‐luciferase reporter kit (Promega) was employed to assess the relative luciferase activity.

### Immunoblot and Immunoprecipitation

Total proteins were extracted and immunoblot analyses were performed. The following antibody were listed: SKP2 (1:1000; NOVUS; NBP3‐16320), p62 (1:500; NOVUS; H00008878‐M01), Keap1 (1:1000; Thermo Fisher Scientific; MA5‐17106), Nrf2 (1:1000; Thermo Fisher Scientific; PA5‐27882), Flag (1:1000; Beyotime; AF5051), Myc (1:1000; Beyotime; AF5054), HA (1:1000; Beyotime; AF5057), and GAPDH (1:1000; Thermo FisherScientific; PA1‐987). GC lysate was combined with 1 µg of the corresponding antibody and incubated on a shaker at 4 °C for 1 h. Then, 20 µL protein‐A‐coated agarose beads (Santa, sc‐2001) were added into cell lysate overnight at 4 °C, followed by immunoblots.

### miRNA Transfection

According to the supplier's instructions, cells were transfected with miR‐1246 mimics, inhibitors, or negative control (NC) chemical synthesis oligonucleotides (Tsingke Biotechnology Co., Ltd., China) at a final concentration of 50 nm using Lipofectamine 3000 reagent (Invitrogen, USA). The sequences for miRNA mimics and inhibitors are listed as follows (5′‐3′): miR‐1246 mimics: AAUGGAUUUUUGGAGCAGG; miR‐1246 inhibitor: CCUGCUCCAAAAAUCCAUU.

### Plasmids Construction

SKP2 (NCBI accession number: NM_001285980.1) and p62 (NM_001290769.1) coding sequences were inserted into pCMV‐Flag and pCMV‐Myc vectors respectively. Primers are listed as follows: Flag‐SKP2: F‐ATATGGATCCATGGGTGTCTCGGCCTTGGAGAAGG, R‐TATACTCGAGTAGACAACTGGGCTTTTGCAGA; Myc‐p62: F‐ATATGAATTCTTATGGCGTCGTTCACGGTGAAGG, R‐TATACTCGAGTCACAATGGTGGAGGGTGCTTCGAA. The restriction sites were indicated by an underline. SKP2 and p62 target siRNAs were chemically synthesized by Ribobio (Guangzhou, Guangdong, China). The Lipofectamine RNAiMAX reagent (Thermo Fisher Scientific's) was used in accordance with the manufacturer's instructions for siRNA transfection. The siRNA sequences are listed as follows: si‐SKP2: AUCACAAAGUCUUUGUCACUC; si‐p62: UGUAGAUGCGGAAGAUGUCAU. The wild‐type ubiquitin and other mutations were obtained from Addgene.

### Generation of CRISPR‐Cas9‐Based Knockout Cells

MiR‐1246 KO cells were produced utilizing the CRISPR‐Cas9 method. After Bbs I digestion, sgRNAs were designed (miR‐1246 KO‐sgRNA: ATAGGTTGATTGCTAGCCTA) and ligated into the pSpCas9 (BB)‐2A‐Puro (PX459) plasmid. GCs were transfected with the recombinant using Lipo 3000 Transfection Reagent (Invitrogen). Puromycin (3 g mL^−1^) was utilized for the screening of individual colonies 48 h after transfection. After removing half of the cells for western blotting analysis, the remaining cells were used for limiting dilution to create a cell clone.

### Statistical Analysis

Statistical analysis was conducted using GraphPad Prism 5 software. The data are presented as mean ± standard error of the mean (SEM). The difference between the control and the experimental groups was analyzed using ANOVA and *t*‐test. A *p*‐value of ≤0.05 was considered statistically significant.

## Conflict of Interest

The authors declare no conflict of interest.

## Author Contributions

H.W., X.L., and Y.X. conceived and designed the study. H.W., Y.X., X.L., B.L., S.X., Z.S., B.F., R.T., R.Y., Q.L., J.Y., D.G., Z.C., and Y.D. performed the experiments. H.W. and Y.X. analyzed the data. H.W., Y.X., and X.L. wrote the manuscript. All authors read and approved the final manuscript.

## Supporting information



Supporting Information

## Data Availability

Data sharing is not applicable to this article as no new data were created or analyzed in this study.
