# Supplementary material for: Bacteroides Fragilis Transplantation Reverses Reproductive Senescence by Transporting Extracellular Vesicles Through the Gut‐Ovary Axis
Source: Adv Sci (Weinh). 2025 Jan 13;12(9):2409740. doi: 10.1002/advs.202409740 (PMC11884595; doi:10.1002/advs.202409740)
Supplement: Supplementary file 1 — Supporting Information [file ADVS-12-2409740-s001.docx]

**Supporting Information**

**Figure Legends**

**Supplementary Figure S1 The difference in ovarian function and microbial profile between aging and young mice**

(**A-C**) Concentrations of E2, FSH and AMH in young and aging mice were tested by ELISA (n=6 in each group; T-test). ***, p < 0.001; **, p < 0.01; *, p < 0.05. (**D**) The number of follicles at different development stages (n=6 in each group; two-way ANOVA). *, p < 0.05. (**E**) The number of oocytes retrieved, fertilized zygotes, cleaved embryos, and blastocysts in different groups (n=6 in each group; two-way ANOVA). *, p < 0.05. (**F**) Relative abundance of BF was test by RT-qPCR (n=6 in each group; T-test). *, p < 0.05.

**Supplementary Figure S2 Mouse gut microbiota profile after treatment**

(**A**) The depletion efficiency of gut microbiota in mice treated with ABX. Control and germ-free mice were used as positive and negative controls, respectively. (**B**) The level of BF in fecal was quantified by RT-qPCR (n=6 in each group; one-way ANOVA). ***, p < 0.001. (**C**) RT-qPCR detected the quantity of BF in young and aging mice feces after BF transplantation (n=6 in each group; one-way ANOVA). *, p < 0.05. (**D**) Cell vitality was evaluated by CCK-8 assay (n=6 in each group; one-way ANOVA). ns, not significant.

**Supplementary Figure S3 *E. coli* and *L. lactis* fail to alleviate ovarian aging**

(**A**) Schematic diagram demonstrating the study design of *in vivo* experiments. (**B-D**) The concentrations of E2, FSH and AMH after *E. coli* or *L. lactis* treatment were tested by ELISA (n=6 in each group; two-way ANOVA). (**E**) Histopathologic images of ovaries in Control, *E. coli*, and *L. lactis* groups. Scale bar, 250 mm. (**F**) The number of follicles at different development stages (n=6 in each group; two-way ANOVA). ns, not significant. (**G**) The number of oocytes retrieved, fertilized zygotes, cleaved embryos, and blastocysts in Control, *E. coli*, and *L. lactis* groups (n=6 in each group; two-way ANOVA). ns, not significant. (**H**) The number of viable off-springs conceived (n=8 in each group; one-way ANOVA). ns, not significant. (**I**) RT-qPCR was performed to determine miRNA levels in liver tissue (n=6, T-test). *, p < 0.05.

**Supplementary Figure S4 BF-EV relieve oxidative stress**

**(A)** Quantification of the number of bacterial colonies in different groups (n=6 per group; one-way ANOVA). ns, not significant. (**B** and **C**) Total protein contents (**B**) and particle numbers of EVs (**C**) from different groups (n=6 per group; one-way ANOVA). ***, p < 0.001; ns, not significant. (**D**) Schematic diagram of the experimental protocol. (**E**) EVs were collected from cell conditioned media and quantified (n=6 in each group, one-way ANOVA). ***, p < 0.001. (**F** and **G**) ROS fluorescence staining (green) (**F**) and relative fluorescence intensity ratio (**G**) (n=28, 28, 30, 29. one-way ANOVA). ***, p < 0.001; ns, not significant. Scale bar, 20 μm. (**H**) GSSG/GSH ratio (n=6 in each group, one-way ANOVA). ***, p < 0.001; ns, not significant. (**I**) The number of oocytes retrieved, fertilized zygotes, cleaved embryos, and blastocysts in Control-IECs-EVs, EVs-IECs-GW4869, BF-IECs-EVs, and BF-IECs-GW4869 groups (n=6 in each group; two-way ANOVA). ***, p < 0.001; **, p < 0.01; ns, not significant. (**J**) The number of viable off-springs conceived (n=8 in each group; one-way ANOVA). ***, p < 0.001; **, p < 0.01; ns, not significant. (**K**) The mRNA expression level of Nrf2 was analyzed in healthy individuals and ovarian cancer patients using data downloaded from the TCGA database.

**Supplementary Figure S5 EVs derived from BF-treated CT26 cells alleviate ovarian aging in a mouse co-culture model**

(**A**) A schematic overview of GCs co-cultured with CT26 cells, which were pre-treated with PBS or BF. (**B**) EVs were collected from conditioned media and quantified. (**C**) Cell vitality was evaluated by CCK-8 assay (n=6 in each group; one-way ANOVA). ns, not significant. (**D**) GSSG/GSH ratio (n=6 in each group, one-way ANOVA). **, p < 0.01; *, p < 0.05. (**E**) Mitochondrial activities were measured using MitoTracker Red (scale bar, 60 μm). (**F**) Relative fluorescence intensity of mitochondrial activities in Control, BF, GW4869, and BF + GW4869 groups (n=6 in each group, one-way ANOVA). ***, p < 0.001. (**G**) OCR measurements were obtained. (**H**) RT-qPCR was performed to determine mRNA levels of oxidative stress-related genes, including SOD1, SOD2, and CAT (n=6 in each group, two-way ANOVA). ***, p < 0.001; **, p < 0.01; *, p < 0.05.

**Supplementary Figure S6 EVs can deliver miR-1246 into ovaries to relieve ovarian aging**

(**A**) Schematic diagram of the experimental protocol. (**B**) RT-qPCR quantification of miRNA levels in EVs obtained from HCT116 cells (WT, miR-1246 KO) as stated in the materials and methods section (n=6 in each group; one-way ANOVA). ***, p < 0.001. (**C-E**) The levels of E2, FSH and AMH were measured by ELISA in different groups (Control-EVs, WT-EVs, and miR-1246 KO-EVs) (n=6 in each group; one-way ANOVA). ***, p < 0.001; **, p < 0.01. (**F**) Histopathologic images of ovaries in Control-EVs, WT-EVs, and miR-1246 KO-EVs groups. Scale bar, 250 mm. (**G**) The number of follicles at different development stages in Control-EVs, WT-EVs, and miR-1246 KO-EVs groups (n=6 in each group; two-way ANOVA). ***, p < 0.001; **, p < 0.01; *, p < 0.05; ns, not significant. (**H**) The number of oocytes retrieved, fertilized zygotes, cleaved embryos, and blastocysts in Control-EVs, WT-EVs, miR-1246 KO-EVs groups (n=6 in each group; two-way ANOVA). ***, p < 0.001; *, p < 0.05; ns, not significant. (**I**) The number of viable off-springs conceived (n=8 in each group; one-way ANOVA). ***, p < 0.001.

**Supplementary Figure S7 SKP2 inhibits p62 expression through ubiquitination modification**

(**A**) After GCs were treated with or without miR-1246 enriched-EVs (WT, si-SKP2), dual luciferase reporter assay was used to analyze the p62 promoter activities (n=6 in each group; one-way ANOVA). (**B**) After treating with or without miR-1246 enriched-EVs into GCs (WT, si-SKP2), p62 mRNA level was determined by RT-qPCR (n=6 in each group; one-way ANOVA). (**C**) GCs (WT, si-SKP2) were treated with 25 μM MG132 for 2 h, followed by treatment with or without miR-1246 enriched-EVs for 24 h, and p62 expression was analyzed by western blotting. (**D**) GCs (WT, si-SKP2) were treated with 10 μM E64d and PepA for 2 h, followed by treatment with or without miR-1246 enriched-EVs for 24 h, and western blotting analysis was used to evaluate p62 expressions. (**E**) GCs (WT, si-SKP2) were treated with 25 μM MG132 for 2 h, followed by treatment with or without miR-1246 enriched-EVs for 24 h. Ubiquitination of p62 was analyzed in WT and si-SKP2 GCs. Blots were representative of three independent experiments.

**Supplementary Figure S8 p62 phosphorylation caused by miR-1246 enriched-EVs treatment promotes Keap1-Nrf2 dissociation and forms p62-Keap1 complex**

(**A**) Western blotting analysis was used to evaluate the protein levels of SKP2, p62, Keap1 and Nrf2 following treatment with miR-1246 enriched-EVs in GCs. (**B**) The endogenous interaction between Keap1, p62 or Nrf2 was examined with or without miR-1246 enriched-EVs treatment. (**C**) Ubiquitination of Nrf2 was analyzed in GCs. (**D**) GCs were treated with miR-1246 enriched-EVs for 24 h, and western blotting analysis was used to evaluate the phosphorylation levels of p62. (**E**) Immunoassay using lysates of GCs cotransfected with vectors expressing Flag-Keap1 and Myc-p62 or Myc-p62 S351A. Blots were representative of three independent experiments.

**Supplementary Figure S1 The difference in ovarian function and microbial profile between aging and young mice**

**
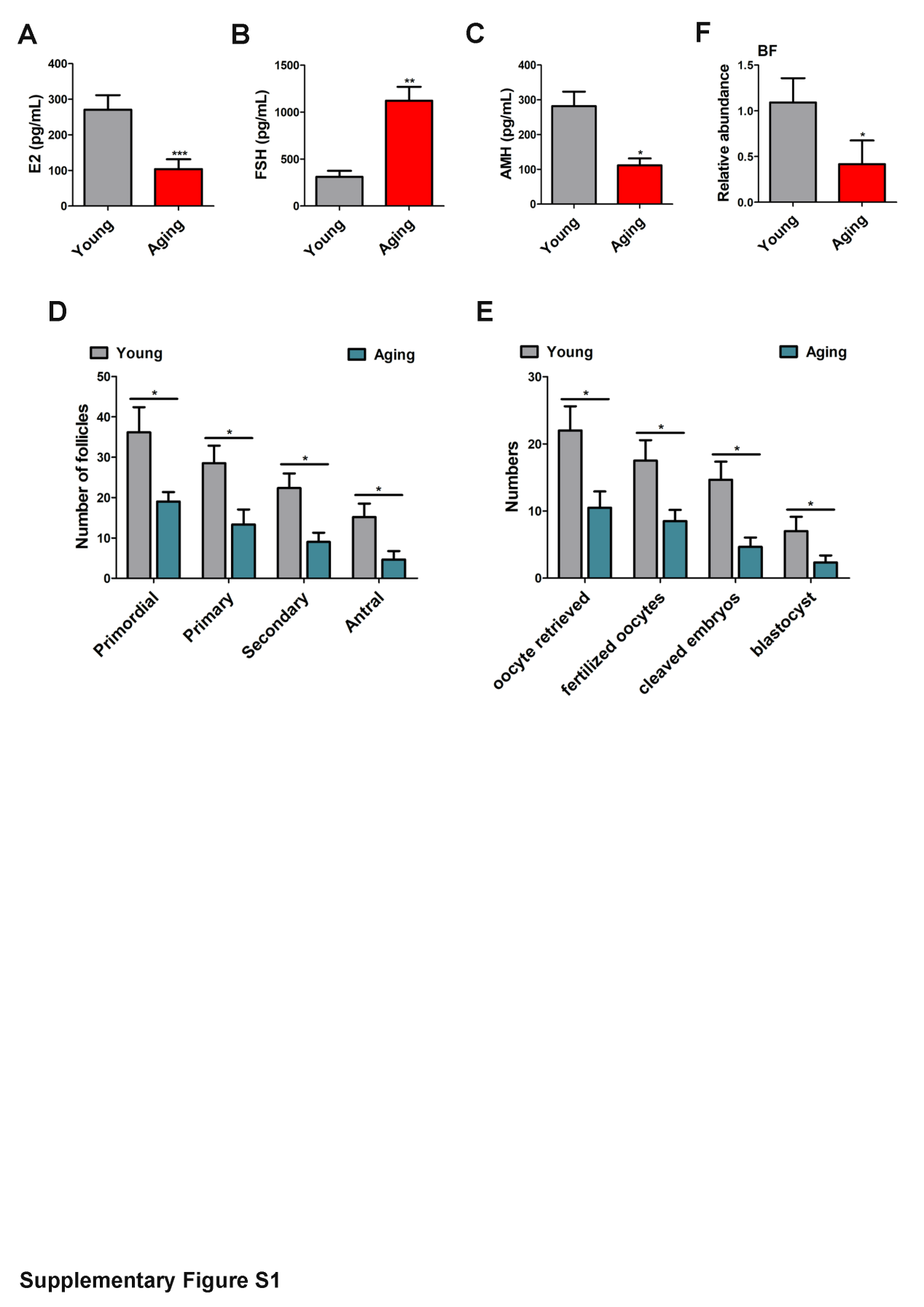
**

**Supplementary Figure S2 Mouse gut microbiota profile after treatment**

**
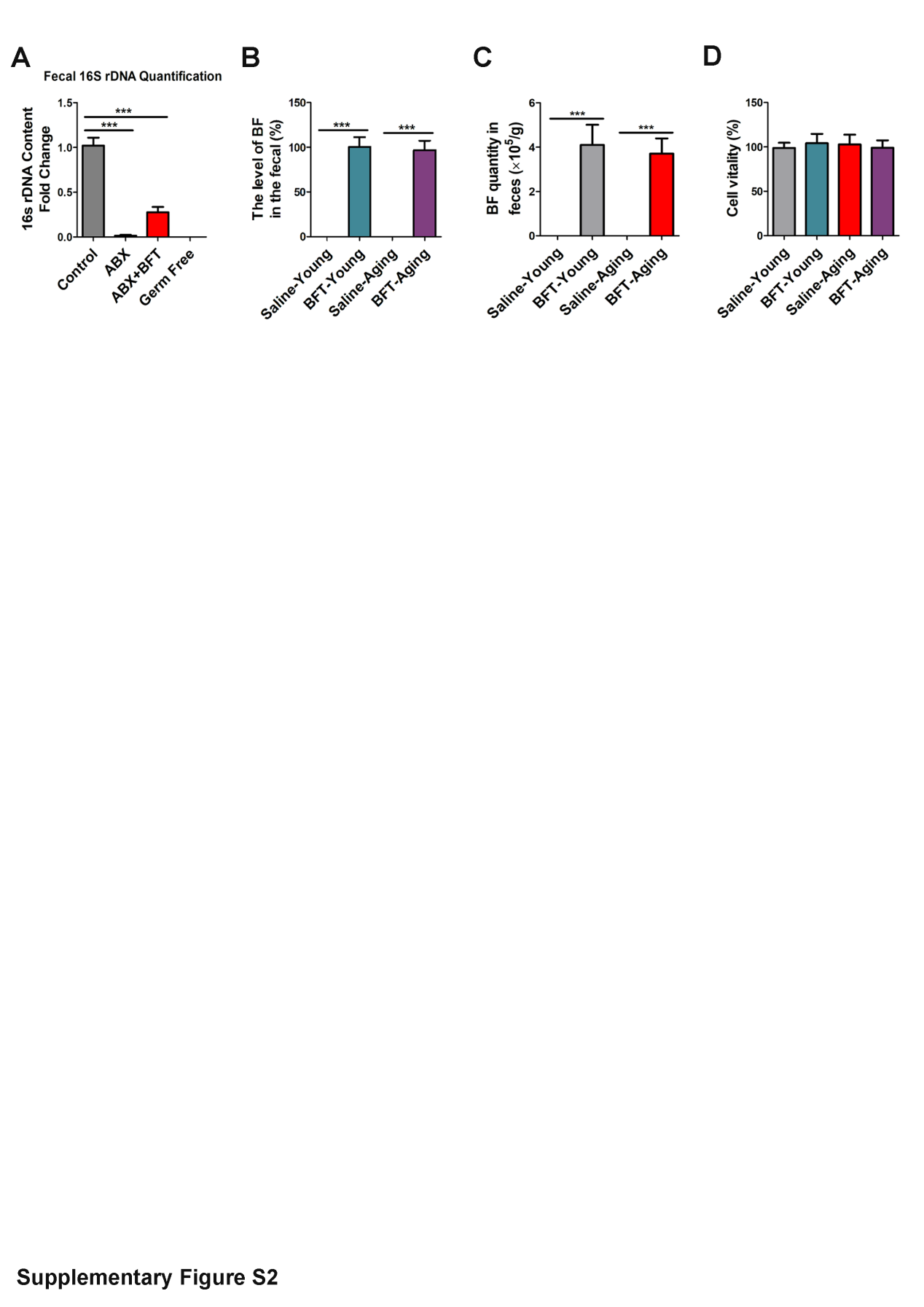
**

**Supplementary Figure S3 *E. coli* and *L. lactis* fail to alleviate ovarian aging**

**
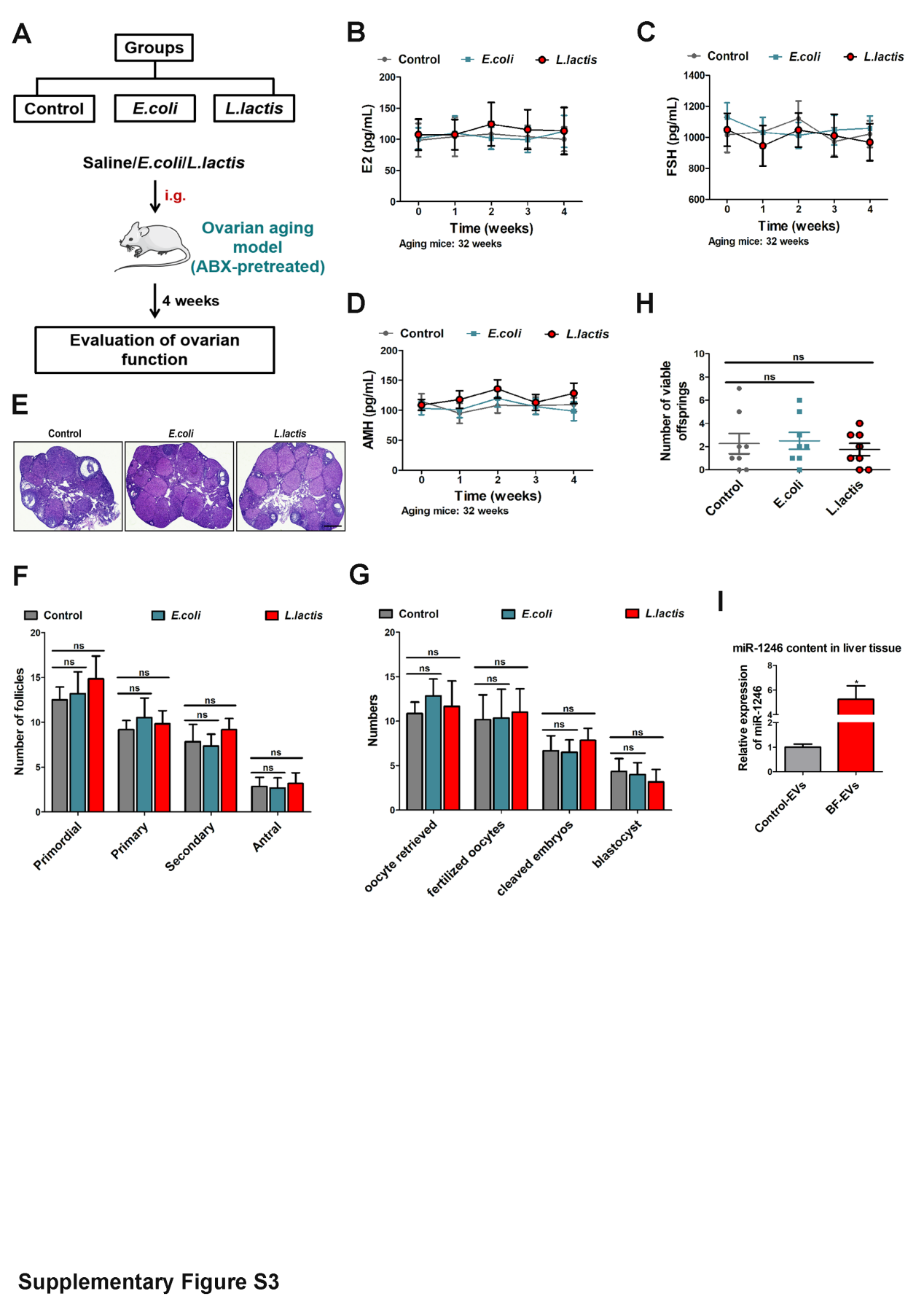
**

**Supplementary Figure S4 BF-EV relieve oxidative stress**

**
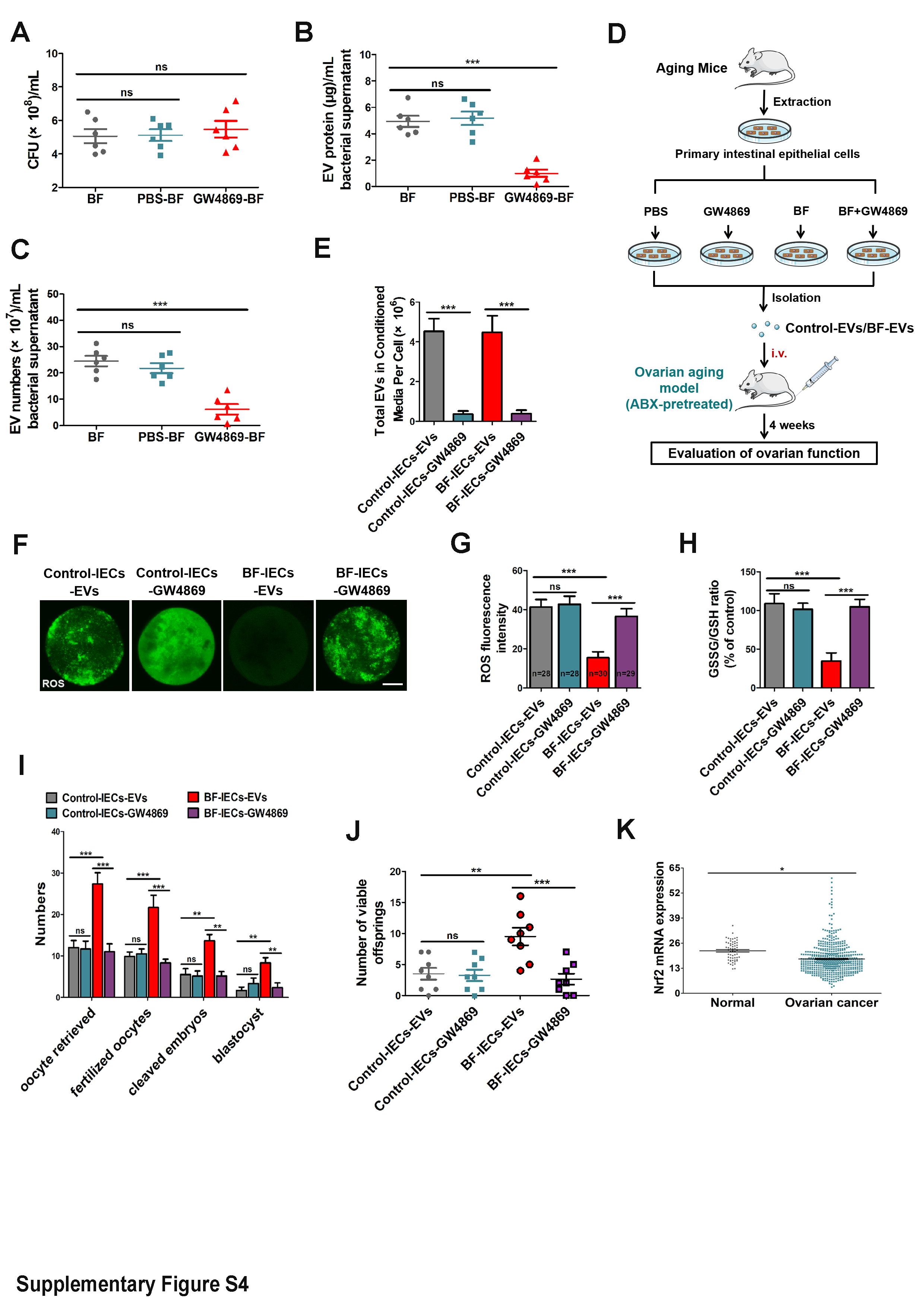
**

**Supplementary Figure S5 EVs derived from BF-treated CT26 cells alleviate ovarian aging in a mouse co-culture model**

**
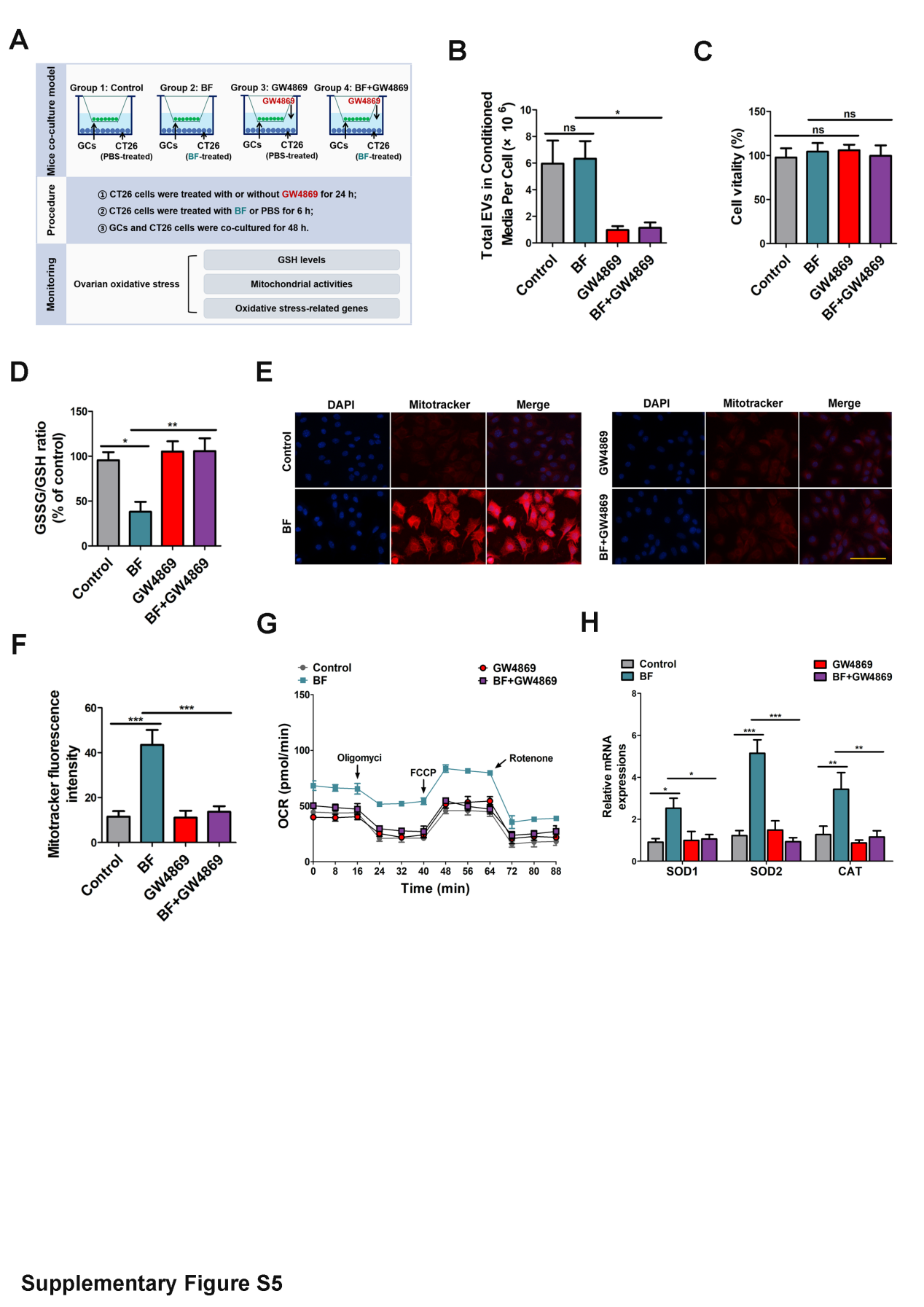
**

**Supplementary Figure S6 EVs can deliver miR-1246 into ovaries to relieve ovarian aging**

**
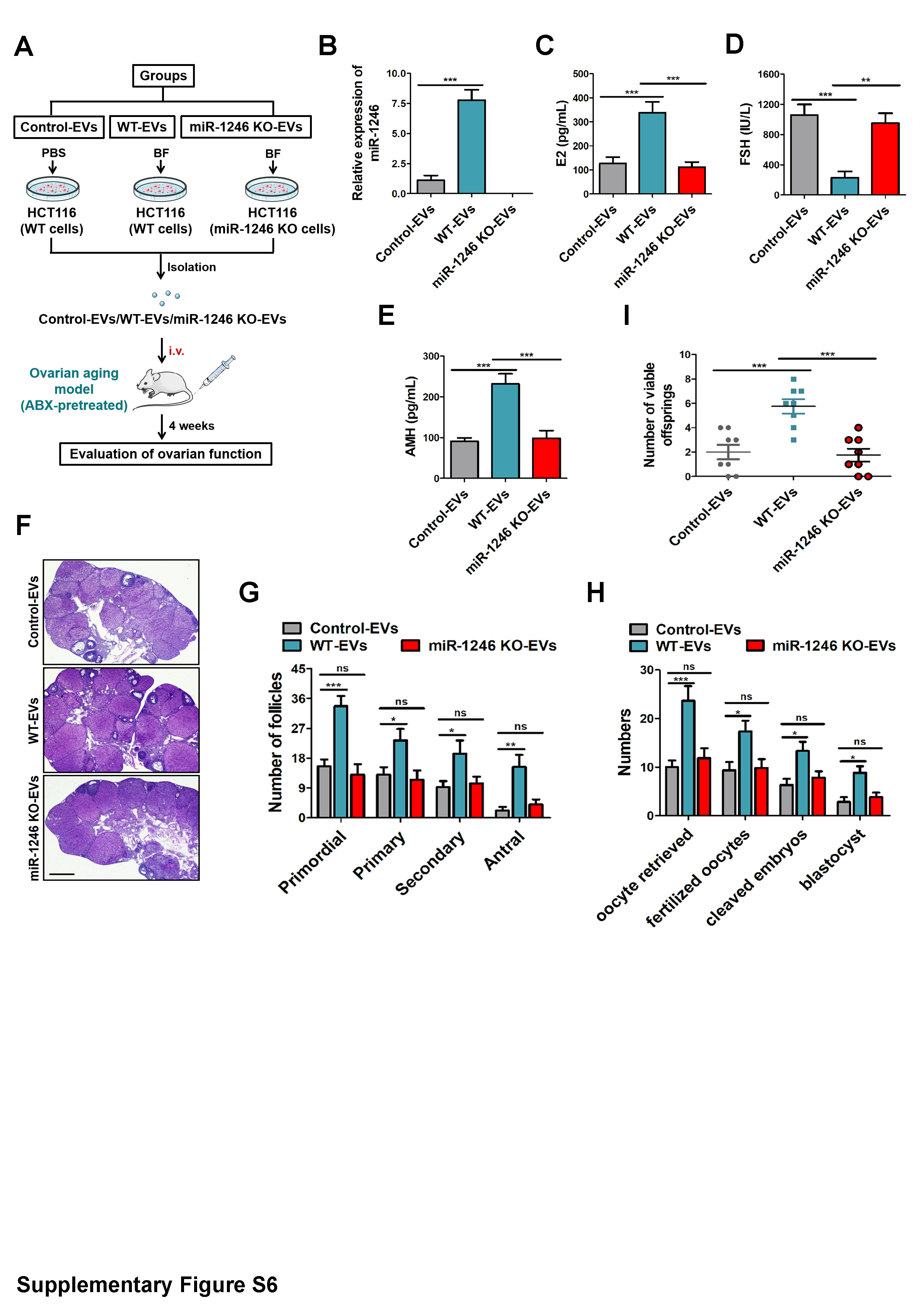
**

**Supplementary Figure S7 SKP2 inhibits p62 expression through ubiquitination modification**

**
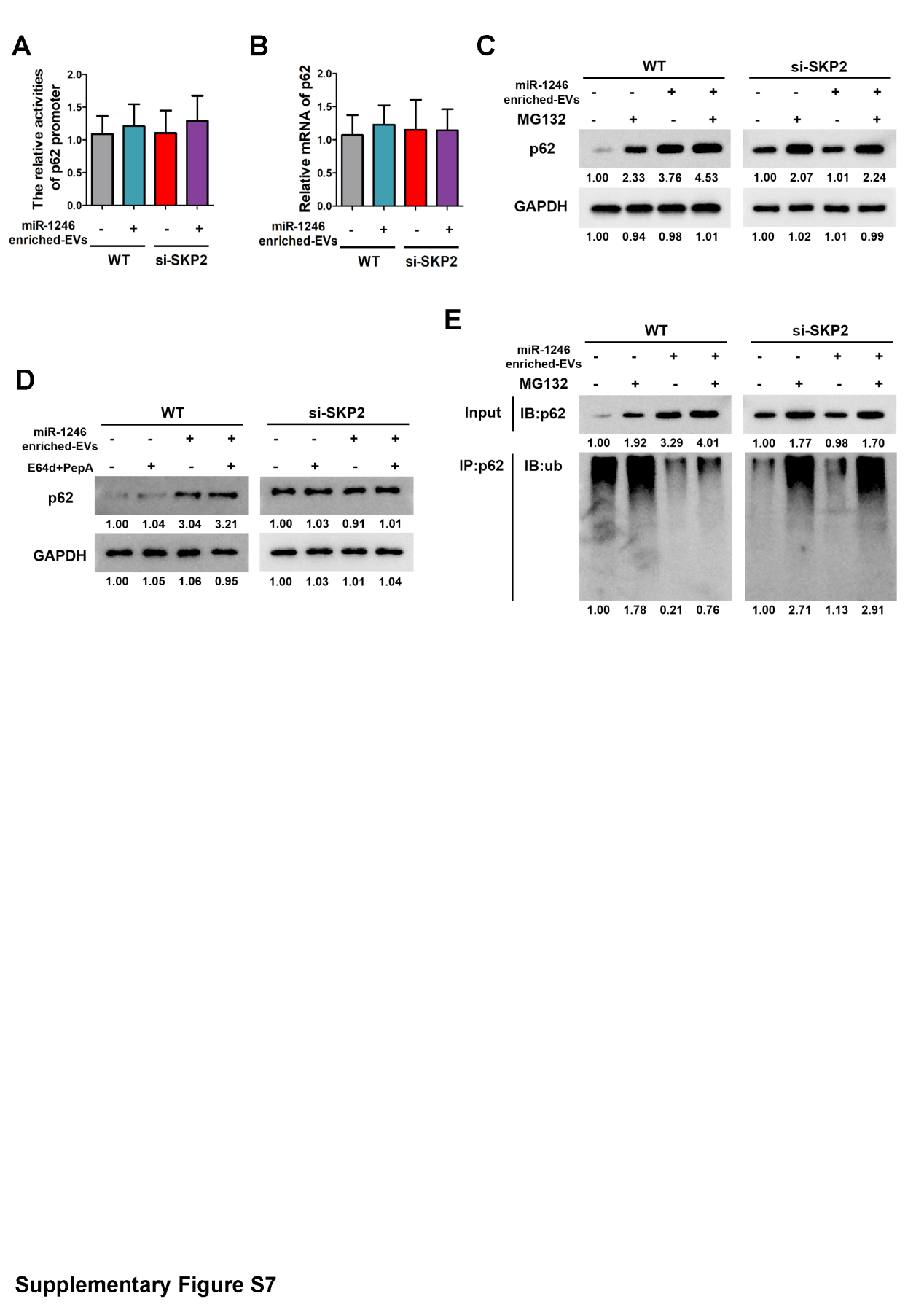
**

**Supplementary Figure S8 p62 phosphorylation caused by miR-1246 enriched-EVs treatment promotes Keap1-Nrf2 dissociation and forms p62-Keap1 complex**

**
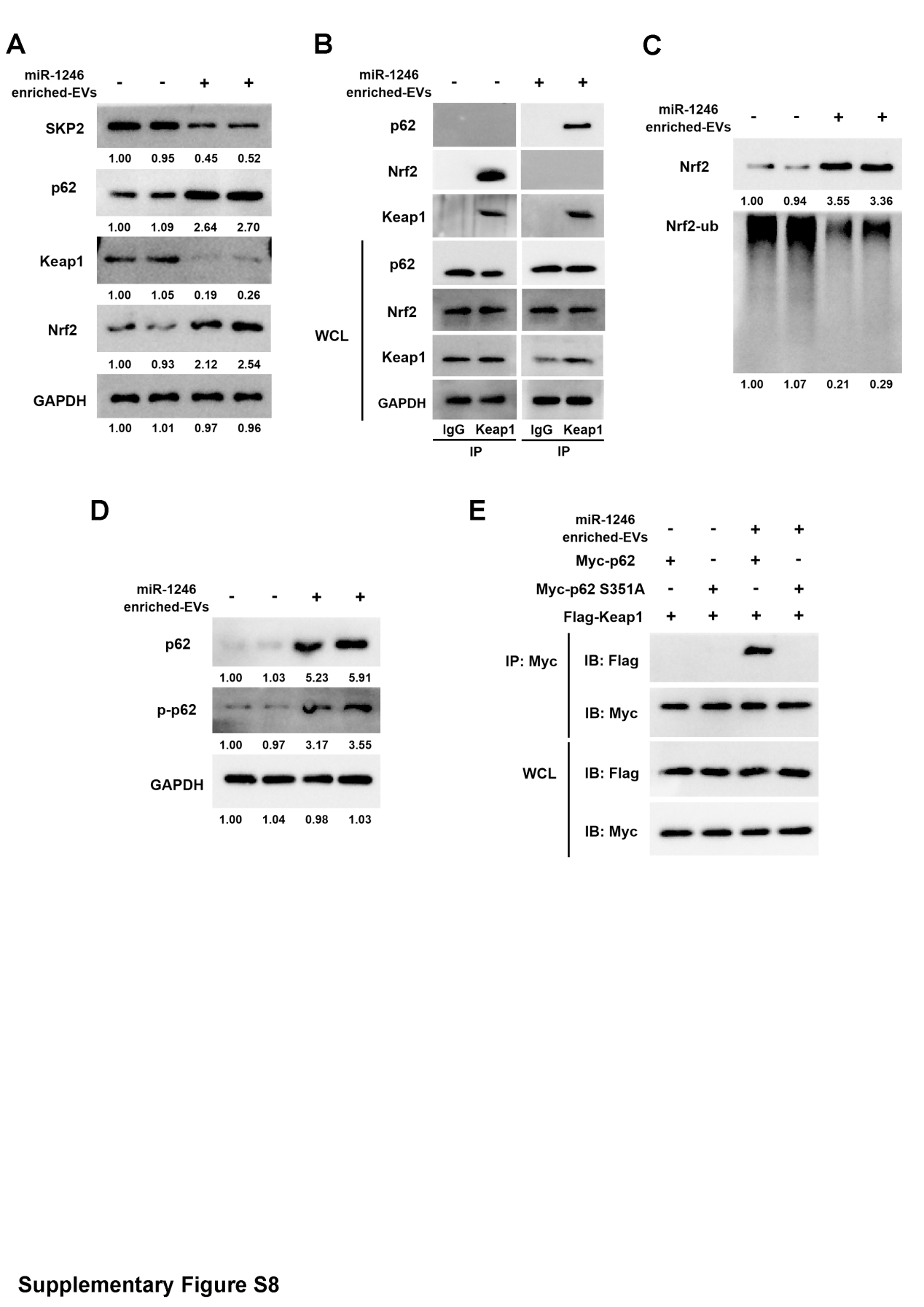
**
